# RefShannon: A genome-guided transcriptome assembler using sparse flow decomposition

**DOI:** 10.1371/journal.pone.0232946

**Published:** 2020-06-02

**Authors:** Shunfu Mao, Lior Pachter, David Tse, Sreeram Kannan

**Affiliations:** 1 Department of Electrical and Computer Engineering, University of Washington, Seattle, WA, United States of America; 2 Division of Biology and Biological Engineering, Caltech, Pasadena, CA, United States of America; 3 Department of Electrical Engineering, Stanford University, Stanford, CA, United States of America; University of Western Sydney, AUSTRALIA

## Abstract

High throughput sequencing of RNA (RNA-Seq) has become a staple in modern molecular biology, with applications not only in quantifying gene expression but also in isoform-level analysis of the RNA transcripts. To enable such an isoform-level analysis, a transcriptome assembly algorithm is utilized to stitch together the observed short reads into the corresponding transcripts. This task is complicated due to the complexity of alternative splicing - a mechanism by which the same gene may generate multiple distinct RNA transcripts. We develop a novel genome-guided transcriptome assembler, RefShannon, that *exploits the varying abundances* of the different transcripts, in enabling an accurate reconstruction of the transcripts. Our evaluation shows RefShannon is able to improve sensitivity effectively (up to 22%) at a given specificity in comparison with other state-of-the-art assemblers. RefShannon is written in Python and is available from Github (https://github.com/shunfumao/RefShannon).

## Introduction

In many higher organisms including mammals, the genomic DNA is comprised of thousands of genes, along with other sequences that regulate when, where and how the genes are produced. These genes have distinct regions called exons and introns [1, 2]. By combining its exons in various ways—a procedure called alternative splicing [3–5], a gene, especially in eukaryotes, can produce multiple different messenger RNAs (mRNA [6], or RNA transcript in this document) that can be converted into different protein products in cells.

Transcriptome is the set of RNA transcripts. With RNA sequencing technology [7], we can now obtain millions of short RNA fragments (RNA-seq reads) from the transcriptome. The transcriptome assembly [8] problem is to obtain a complete and accurate recovery of transcriptome based on observed RNA-seq reads. This helps us find new RNA transcripts as well as their expression levels (or abundance) in order to better understand proteins and cells.

Transcriptome assembly is not an easy task due to several factors. Because of alternative splicing, it is possible that different set of transcripts can yield the same observation of RNA-seq reads. Adding to this complexity is the fact that distinct transcripts are expressed at different expression levels [9]. Thus transcripts which have low expression levels are harder to reconstruct; also, transcripts which are part of complex isoform [4] families are difficult to assemble correctly.

There are two flavors of the transcriptome assembly problem [8]: de novo assembly and genome-guided (or reference-based) assembly. For de novo assembly, there is no knowledge other than that of the observed reads. This is common in non-model organisms or when an approach unbiased by prior knowledge is needed (e.g. cancer transcriptome). For genome-guided assembly, in addition to the observed reads there is also knowledge of the genome of the organism. This is common in scenarios where a model organism [10] is sequenced.

De novo assembly is typically more challenging and less accurate than genome-guided assembly, since the latter utilizes additional side information. While algorithms and software packages are available for both the de novo (TransAByss [11], Trinity [12], OASES [13], SOAPdenovo-Trans [14] etc) and genome-guided assembly problems (Scripture [15], Cufflinks [16], StringTie [17], TransComb [18] and CLASS2 [19], Ryuto [20], Strawberry [21], Trinity (reference-guided mode) [12] etc), much remains to be done [22, 23]. Recently Kannan et al [24] developed an assembler called Shannon assembler that utilized principles from information theory to solve the de novo transcriptome assembly problem, and demonstrated benefits over state-of-the-art assemblers. Here we develop a genome-guided assembler called RefShannon by exploiting the framework from Shannon.

To begin with we note that, while genome-guided algorithms have an advantage in general, Shannon is able to recover even more transcripts than the leading genome-guided assembler StringTie when the coverage of the transcripts is high (S1 File). We attribute this to the careful utilization of transcript abundances while performing assembly in Shannon. However, as expected, for transcripts of low abundance, Shannon is inferior because the k-mer graph utilized by Shannon needs a higher coverage in order to stay connected. Therefore, it should be possible to design a genome-guided assembler, that combines the superior reconstruction method of Shannon along with the aid of the genome side-information in order to deliver optimal performance. This is the main motivation for this work—to build a superior genome-guided assembler.

## Results

In this section, we will describe the overall work flow of RefShannon, and highlight its main ideas. To demonstrate its superior performance, we have compared RefShannon to two widely used assemblers StringTie [17] and Cufflinks [15], which are recommended in previous benchmark work [23]. We have also compared RefShannon to guided Trinity [12] and recently published Ryuto [20], as they show relatively good performance among various assemblers in our initial analysis using smaller datasets (S3 File). Our performance evaluation metrics include ROC (including sensitivity and false positive) for simulated datasets and sensitivity for real datasets. Lastly, we also discuss its computational complexity.

### Overall workflow

To do genome-guided transcriptome assembly based on sampled RNA-Seq reads, RefShannon takes a graph preparation step and a graph traversal step, as other assembly methods [16–21] usually do.

**Fig 1:**
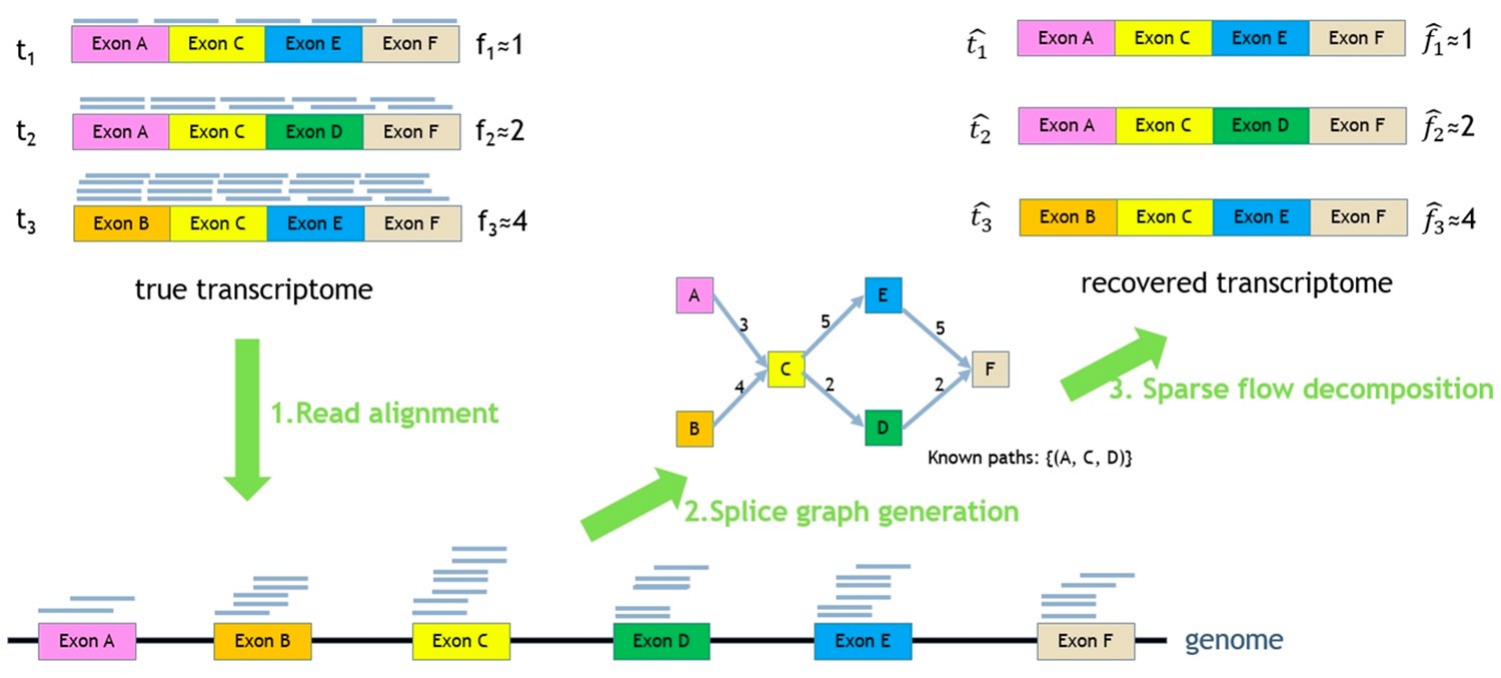
Overall flow of RefShannon. In this example, the true transcriptome contain 3 RNA transcripts *t*_1_, *t*_2_, *t*_3_ with expression levels *f*_1_ = 1, *f*_2_ = 2, *f*_3_ = 4. RNA reads are sampled from the transcriptome first, and are aligned onto a reference genome using external aligners. RefShannon will take the read alignment as input, generate splice graphs, and apply a novel sparse flow decomposition algorithm to recover the transcriptome as $\hat{t_{i}}$ *i* ∈ {1, 2, 3}. The corresponding $\hat{f_{i}}$ estimates related abundance.

In particular as Fig 1 illustrates, RNA-Seq reads sampled from transcriptome will be aligned onto a reference genome using external tools such as STAR [25], Tophat2 [26], Hisat2 [27], GMAP [28], minimap2 [29] and so on. These tools are able to capture splice events (e.g. a read crossing two distinct exons of an RNA transcript can be split aligned onto the genome), which enable us to infer exon regions from read alignments and consequently do not need existing gene annotations for transcriptome assembly.

Based on read alignments, RefShannon produces splice graphs, where each node represents a unique exonic region that is supported by aligned reads and each edge between two nodes implies there exist reads going through those nodes. Additional known paths are also collected, indicating that the nodes along the path belong to some transcript. This helps resolve flow decomposition ambiguity, as will be discussed later.

Based on splice graphs, RefShannon applies a sparse flow decomposition algorithm, originally proposed in [24], to reconstruct the minimum number of flow paths (as assembled transcriptome) that satisfy node and edge constraints.

### Main ideas

**Fig 2:**
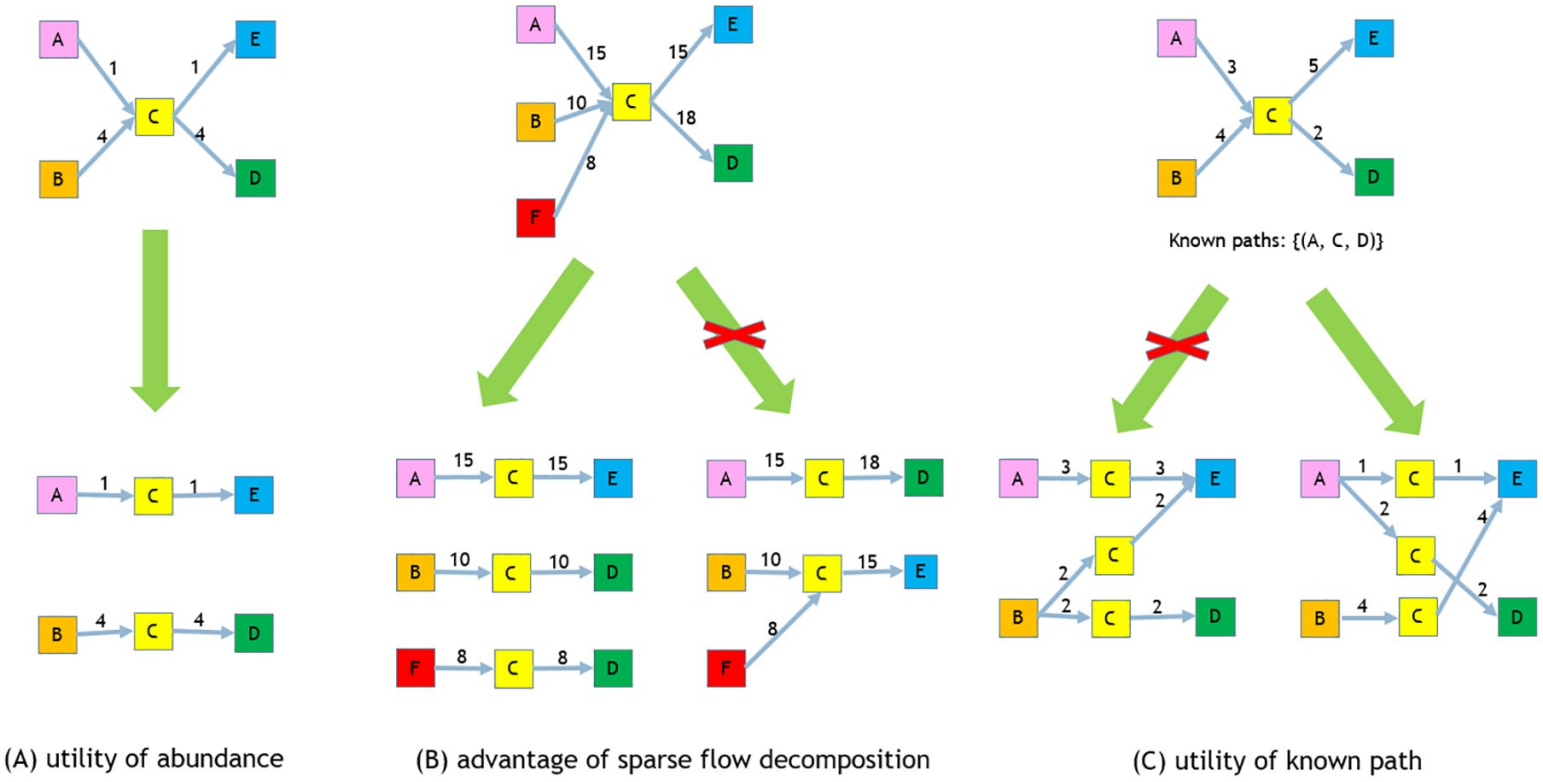
Main ideas of RefShannon. In these conceptual examples, each graph means a splice graph where nodes represent exonic regions and edges indicate there are reads aligned across the nodes. The edge weights are abundance, calculated by the number of supporting reads. (A) RefShannon explores the utility of varying abundance, which is essential for a correct decomposition of paths. For example, given that AC, CE, BC, CD have weights 1, 1, 4, 4 respectively, we’re confident to decompose the graph into two paths as ACE and BCD. (B) RefShannon adopts a sparse flow algorithm that tries to find the minimum number of paths that explains edge weight constraints (e.g. the decomposition of graph into ACE with weight 15, BCD with weight 10 and FCD with weight 8 explain the splice graph well). If we follow a greedy approach (as used by StringTie) that iteratively finds and remove the heaviest path, we will get an inaccurate transcript ACD first. (C) RefShannon extracts known path information from read alignments to resolve decomposition ambiguity. For example, when node C is decomposed, a flow ambiguity (i.e. non unique decomposition) happens: both ACE, BCE, BCD and ACE, ACD, BCE explain node C’s edge weight constraints. Suppose ACD is a known path, then the first decomposition of ACE, BCE, BCD can be excluded.

We would like to highlight several main ideas (as summarized in Fig 2) that distinguish RefShannon from the existing methods (such as Cufflinks [16], StringTie [17], Ryuto [20] and guided Trinity [12] which we’ll mainly compare to) and bring its superior performance.

To begin with, RefShannon takes a graph preparation step and graph traversal step as most existing methods do. The constructed graph is consistent with the reference genome, similar to Cufflinks, Stringtie and Ryuto. Trinity in genome-guided mode also utilizes the reference genome, but mainly to group together the reads within the same region. Essentially it still applies de novo assembly, but to smaller regions. Consequently, guided Trinity is computationally much more complicated (S7 File).

One of the main ideas of RefShannon is to utilize the varying abundance information while traversing the splice graph (Fig 2A). This information is essential for a correct decomposition of flow paths. Cufflinks [16] does not exploit the abundance information during assembly; Instead, it relies on overlap graphs, which require a strict partial order between read alignments. Consequently Cufflinks may throw away reads (especially pair end reads) of uncertain compatibility, while those read alignments can contain useful information to construct a more accurate graph.

Another idea RefShannon has adopted is the sparse flow decomposition algorithm that was initially proposed in the Shannon assembler [24], which applies linear programming to efficiently decompose for the minimum number of paths (flows) at each node restricted by the node’s in-edge and out-edge weights. This approach has been proved in [24] to work toward optimal transcriptome assembly. The similar goal of parsimonious assembly is pursued by Cufflinks [16], which as mentioned earlier is based on overlap graph and does not exploit abundance information. StringTie [17] also explores the abundance information, but it follows a sub-optimal greedy approach by iteratively extracting the heaviest path as transcript. As illustrated in Fig 2B, this will lead to incorrect assembly.

In addition RefShannon has utilized pair end reads to supplement additional edge and path information so that an originally disconnected transcript could be found. A similar idea was also used in Scripture [15] and Ryuto [20]. However Scripture exhaustively enumerates all possible transcript candidates first and filter them later based on certain significance criteria. Ryuto has also adopted these information mainly during the graph construction stage. Differently, RefShannon has also applied these additional edge and path information in the graph decomposition stage so that certain assembly ambiguity can be resolved (Fig 2C). StringTie [17] on the other hand, does not explore these information directly; instead it relies on external software MaSuRCA [30] to generate super reads to fully utilize pair end reads.

### Datasets

Our performance evaluation is based on both simulated datasets and real datasets, as summarized in Table 1.

**Table 1:** Data statistics. There’re three real datasets (HESC—human embryonic stem cells, LC—Lymphoblastoid cells, Kidney) and three simulated datasets (HESC-Sim, LC-Sim, Kidney-Sim) used for evaluation. PE stands for pair-end reads and SE for single-end reads. Oracle Set contains fully covered transcripts from the available reference transcripts.

	HESC	LC	Kidney	HESC-Sim	LC-Sim	Kidney-Sim
PE or SE	SE	PE	PE	SE	PE	PE
Read Length	50	101	100	50	101	100
Num of Reads (SE) or Read Pairs (PE)	132.05M	115.36M	183.53M	150M	150M	150M
Num of Reference Transcripts	13274	207266	207266	207266	207266	207266
Num of Reference Transcripts (Oracle Set)	2694	32394	15605	-	-	-

The real datasets include 132.05M Illumina single end reads (50-bp) sampled from human embryonic stem cells (HESC) (GSE51861 [31]) with 13274 reference transcripts, 115.36M Illuminar pair end reads (101-bp) sampled from Lymphoblastoid cells (LC) (SRP036136 [32]) with 207266 reference transcripts. We also use 183.53M Illuminar pair end reads (100-bp) sampled from HEK293T (Kidney) cells (SRX541227) previously produced and studied in StringTie [17]. The HESC reference transcripts are assembled by hybrid assembler IDP from the 135M short reads together with 7.8M long PacBio reads [31]. The LC reference transcripts are based on GENCODE annotations augmented by utilizing a combination of short reads with long PacBio reads line (700K CCS reads) [32]. We also use LC reference transcripts for Kidney dataset.

The simulated datasets are generated based on the real ones. Specifically, we choose LC reference transcripts as the ground truth transcriptome for all simulated datasets. We then use RSEM [33] to learn parameters from each real dataset based on the alignments of real reads onto the reference transcripts. Based on the gound truth and learned parameters, we finally generate the relevant simulated reads.

### Experiment procedure

The experiment procedure is as follows. For each dataset, we first use STAR aligner [25] to align reads onto reference genome (human genome hg19, downloaded from http://hgdownload.cse.ucsc.edu/goldenpath/hg19/bigZips/) that contains multiple chromosomes. We choose STAR aligner since it offers better performance for most of the assemblers in our evaluation than using alternative aligners such as Tophat2 [26] and Hisat2 [27] (S6 File); in addition different aligners do not affect the comparison conclusions (S3 File). We then apply assemblers onto read alignments to reconstruct transcripts for all chromosomes. The assemblers which we have selected to compare RefShannon to include Cufflinks (v2.2.1), StringTie (v1.3.4d), Ryuto (v1.3m) and Trinity (v2.9.1) as they show relatively good performance in our initial analysis of various assemblers using smaller datasets (S3 File). Finally we evaluate the reconstructed transcripts, and compare them with relevant reference transcripts to see the performance of ROC (including sensitivity and false positive) for simulated datasets and sensitivity for real datasets.

### ROC of simulated datasets

For simulated datasets (HESC-Sim, LC-Sim, Kidney-Sim in Table 1), since we know the ground truth reference transcripts, we check the performance of receiver operating characteristic curves (ROC), which includes sensitivity as well as false positive.

The metric of sensitivity describes how many reference transcripts have been correctly reconstructed. To evaluate sensitivity, we first use blat (https://genome.ucsc.edu/goldenpath/help/blatSpec.html) to create a mapping between reconstructed transcripts *T*_*rec*_ and reference transcripts *T*_*ref*_. We associate each reference transcript *t* ∈ *T*_*ref*_ with a reconstructed transcript *r* ∈ *T*_*rec*_ that mostly matches *t*. We then consider each *t* ∈ *T*_*ref*_ is correctly recovered if it is over 90% matched with its associated *r* ∈ *T*_*rec*_.

The metric of false positive describes how many transcripts are falsely reconstructed and do not belong to the true reference transcripts. Based on the blat result, we consider a reconstructed transcript *r* ∈ *T*_*rec*_ to be a false positive if it is below 90% matched with any reference transcript *t* ∈ *T*_*ref*_. Note if *r* ∈ *T*_*rec*_ is contained in a reference transcript *t* ∈ *T*_*ref*_, it is not considered to be a false positive.

In addition to the threshold of 90%, we have also tried other thresholds, and it does not affect our comparison conclusions (S5 File).

To have a comprehensive understanding of the ROC performance, we run StringTie and Cufflinks in both default mode and max sensitivity mode; we run Ryuto and guided Trinity in their default modes after tuning several parameters which don’t affect sensitivity much (S4 File). We also tune RefShannon so that it is able to adaptively adjust its splice graph generation as well as sparse flow decomposition output in order to trade off its sensitivity and false positive performance (S2 File).

**Fig 3:**
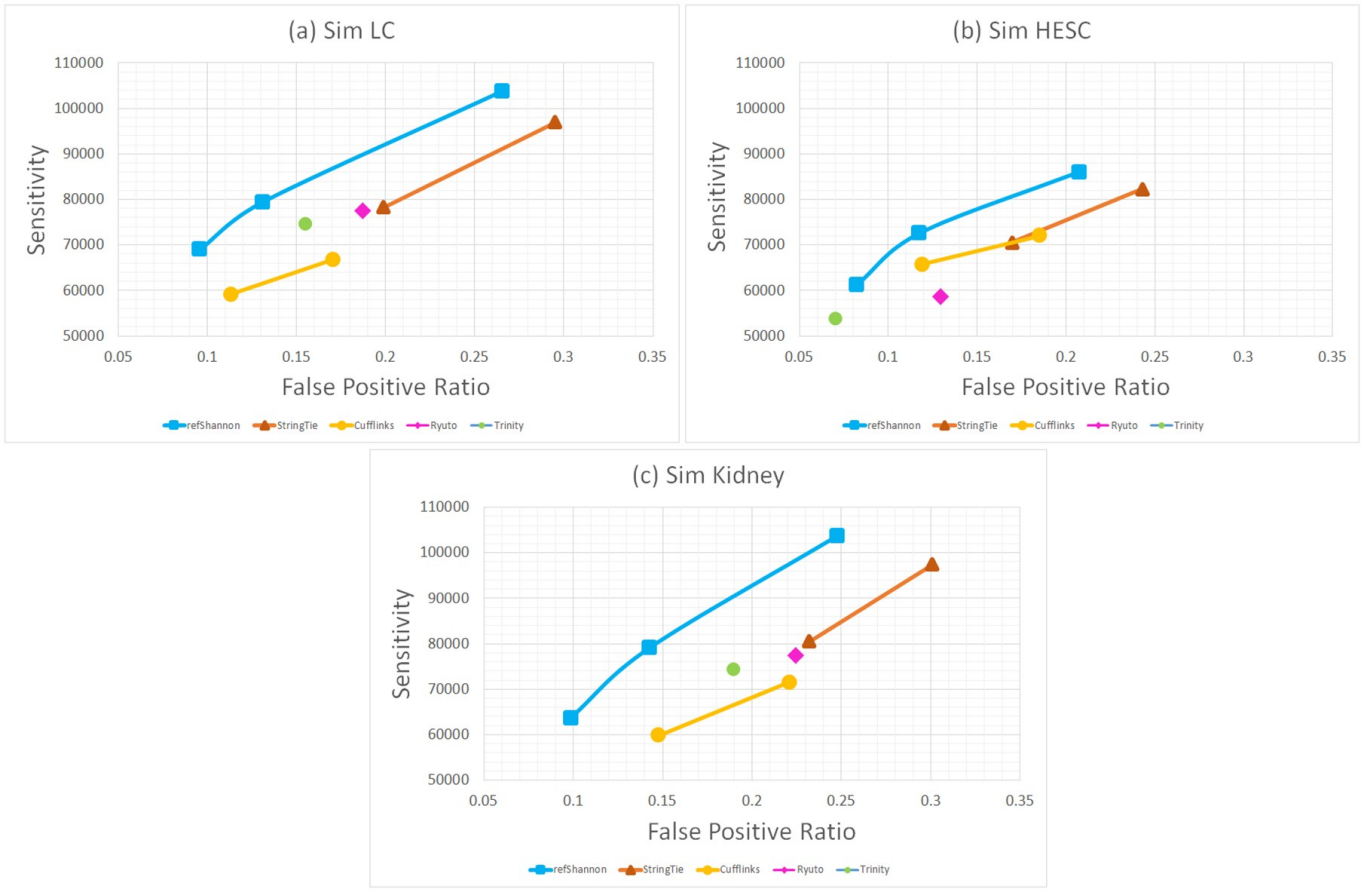
ROC analysis of simulated datasets.

Fig 3 illustrates the ROC performance. Overall RefShannon shows higher sensitivity at a given false positive ratio than other assemblers. For example in simulated LC dataset, the max sensitivity point of RefShannon (the top right blue point) has higher sensitivity and lower false positive than StringTie in max sensitivity setting and the min false positive point of RefShannon (the lower left blue point) has higher sensitivity and lower false positive than Cufflinks in default setting. Quantitatively, if we fix false positive ratio at 15.5%, we obtain a sensitivity gain of 13.5% over guided Trinity which has the second best ROC point. The conclusion is similar in the Kidney dataset, where RefShannon obtains a sensitivity gain of 22% over guided Trinity at 19% false positive ratio. In the HESC dataset, RefShannon shows an ROC trend similar to guided Trinity, and has a sensitivity gain of 10.6% over Cufflinks which shows the next best ROC point at 12% false positive ratio. There is a larger gain in LC and Kidney datasets of pair-end reads than in HESC dataset of single-end reads probably because RefShannon is able to better utilize the pair-end information not only in the splice graph construction but also in the flow decomposition stage.

### Sensitivity of real datasets

For real datasets, since it’s hard to judge if a reconstructed transcript is a false positive one or an unknown transcript yet to be discovered. Therefore, we focus only on sensitivity performance which means among the known reference transcripts, how many of them are correctly recovered.

The sensitivity calculation is similar to previous ROC approach. The difference is (1) For fair evaluation of sensitivity, we run assemblers all in their max sensitivity settings (detailed configurations in S4 File). (2) We only use the oracle set of reference transcripts (statistics in Table 1) for each real dataset. The oracle set contains reference transcripts that are fully covered by reads (tolerated by 25 bp from both ends). We expect these well expressed reference transcripts shall be reconstructed by assemblers.

**Fig 4:**
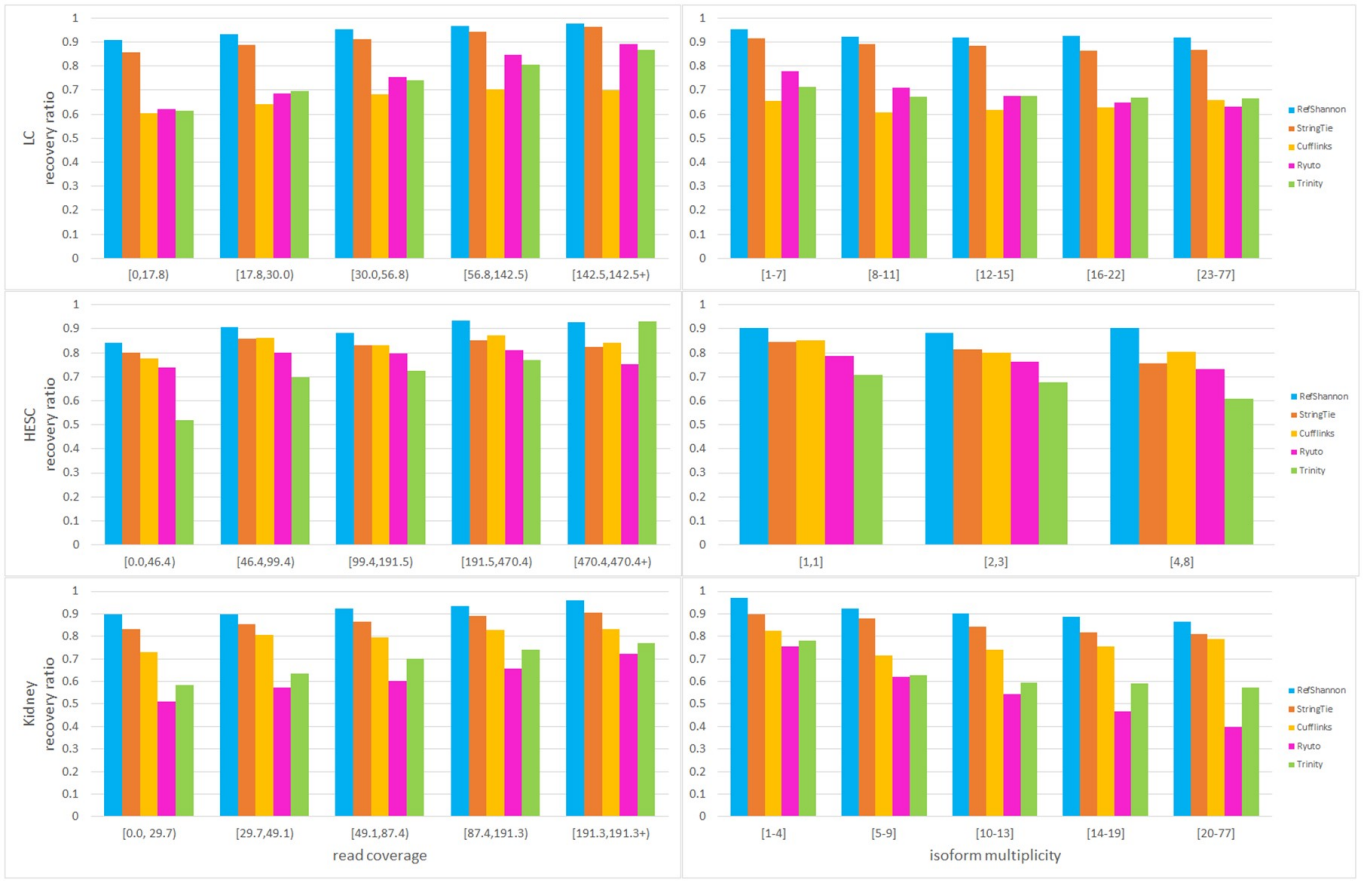
Sensitivity analysis of real datasets. The y-axis of each subplot is recovery ratio, the ratio of the number of correctly reconstructed reference transcripts over the total number of (oracle) reference transcripts. The x-axis of each subplot in left column is read coverage, and the x-axis of each subplot in right column is isoform multiplicity. A lower read coverage implies less expression level of reference transcript in cells, and a higher isoform multiplicity implies more complex splicing patterns. Isoform multiplicity in HESC is lower than that of LC data, implying a simpler splicing structure of reference transcripts in HESC data.

Fig 4 shows our sensitivity evaluation for the three (i.e. LC, HESC, Kidney) real datasets. In the three subplots of first column, reference transcripts in the oracle set are grouped according to their read coverage. If a reference transcript has a low (or high) read coverage, it implies its expression level in cells is low (or high). Note the oracle sets of reference transcripts are different among three datasets, so the group values per dataset are different. For LC and Kidney datasets, most of the reference transcripts are within lower coverage. Therefore, we group the reference transcripts into read coverage of 60, 10, 10, 10 and 10 percentile. For HESC, the reference transcripts are grouped into 20 percentile each. For each dataset under various read coverage conditions, RefShannon has recovered reference transcripts better than all other assemblers. In the three subplots of second column, reference transcripts are grouped according to their isoform multiplicity. Recall that a gene may contain multiple transcripts (i.e. isoforms) due to alternative splicing, and the isoform multiplicity of a transcript refers to the number of isoforms of that transcript’s gene. A higher isoform multiplicity value implies a more complex splice graph for recovery and also implies longer transcript length. Here the reference transcripts for HESC have relatively simple isoform multiplicity (most equals to 1), so we group reference transcripts into about 70, 15 and 15 percentile, while the reference transcripts for LC and Kidney datasets are grouped into 20 percentile each. For each dataset under various isoform multiplicity regions, RefShannon has also recovered more reference transcripts than all other assemblers. Note also that Cufflinks’s performance drops in LC and Kidney compared to HESC, this could be because reads from LC and Kidney datasets are pair end and there are more read alignments Cufflinks may consider to be compatibility uncertain and thus throw away. In addition, guided Trinity does a good job to recover the transcripts of high expression levels in HESC dataset, this could be because the relevant underlying assembly graphs are less fragmented.

### Computation resources

To understand how much time/memory RefShannon requires for assembly tasks, we have monitored assembly procedures using the cgmemtime tool (https://github.com/gsauthof/cgmemtime), which was previously adopted to compare the computational complexities among read aligners [34]. We put details in S7 File. RefShannon is overall faster than guided Trinity, Cufflinks, and Ryuto (for large dataset and more processes). RefShannon consumes more memory compared to other assemblers (except guided Trinity which essentially conducts de novo assembly). This could be because RefShannon is written in Python and memory sharing is less efficient especially for multiprocessing. Currently, a typical lab server with at least 20 CPU cores and over 200GB memory would be sufficient to run RefShannon on large real datasets. One of our future direction is to further improve its computational efficiency.

## Discussions

We have developed RefShannon—a new genome-guided transcriptome assembler as an extension of the original de novo Shannon project [24]. It utilizes a careful splice graph generation procedure aimed at capturing as much information as possible from read alignments, and utilizes a sparse flow decomposition algorithm aims at reconstructing as small number of transcripts as possible under splice graph constraints. Our evaluation shows performance gain of RefShannon over state-of-art genome guided assemblers for both simulated and real datasets. We expect RefShannon will help discover novel genes and isoforms that may be missed by existing transcriptome assemblers. We also expect its intermediately generated splice graphs (with nodes, edges and paths) will provide helpful interface to be used by other relevant research.

There are several future directions. One of them is to further improve the computational efficiency as described previously.

In addition, it shall be useful to incorporate the third generation long reads (e.g. PacBio [35] and Nanopore reads [36]) to assist the short read assembly process. Compared to the short RNA reads (e.g. <300 bp), long reads (e.g. >10000 bp) will be able to better bridge multi-exons and resolve repetitive regions. However they are more expensive (e.g. 100 to 280 Euro per isolate) and have higher errors (typically 10% to 15%), whereas short reads are more cost-effective (e.g. 40 Euro per isolate) and also much more accurate (typically below 0.1% error rate) [37, 38]. Ideally, applying long contigs (or reads) into short read assemblers could be an effective feature [17, 20].

Moreover, it could be interesting to apply RefShannon onto the downstream analysis such as variant calling (e.g. SNP or small indels). RefShannon shows a better sensitivity (under a similar false positive rate) than existing assemblers, which implies additional new RNA transcripts could be discovered. Using variant callers (e.g. GATK [39] or recently developed abSNP [40]) to look for variants from newly discovered transcripts may help scientists gain new medical insights.

## Methods

As the overall work flow is described in Results, in this section, we will describe the graph generation and traversal steps of RefShannon.

### Splice graph generation

The splice graph generation consists of three steps: split, merge and connect. The pseudo code can be obtained from Algorithm 1 in S2 File.

**Fig 5:**
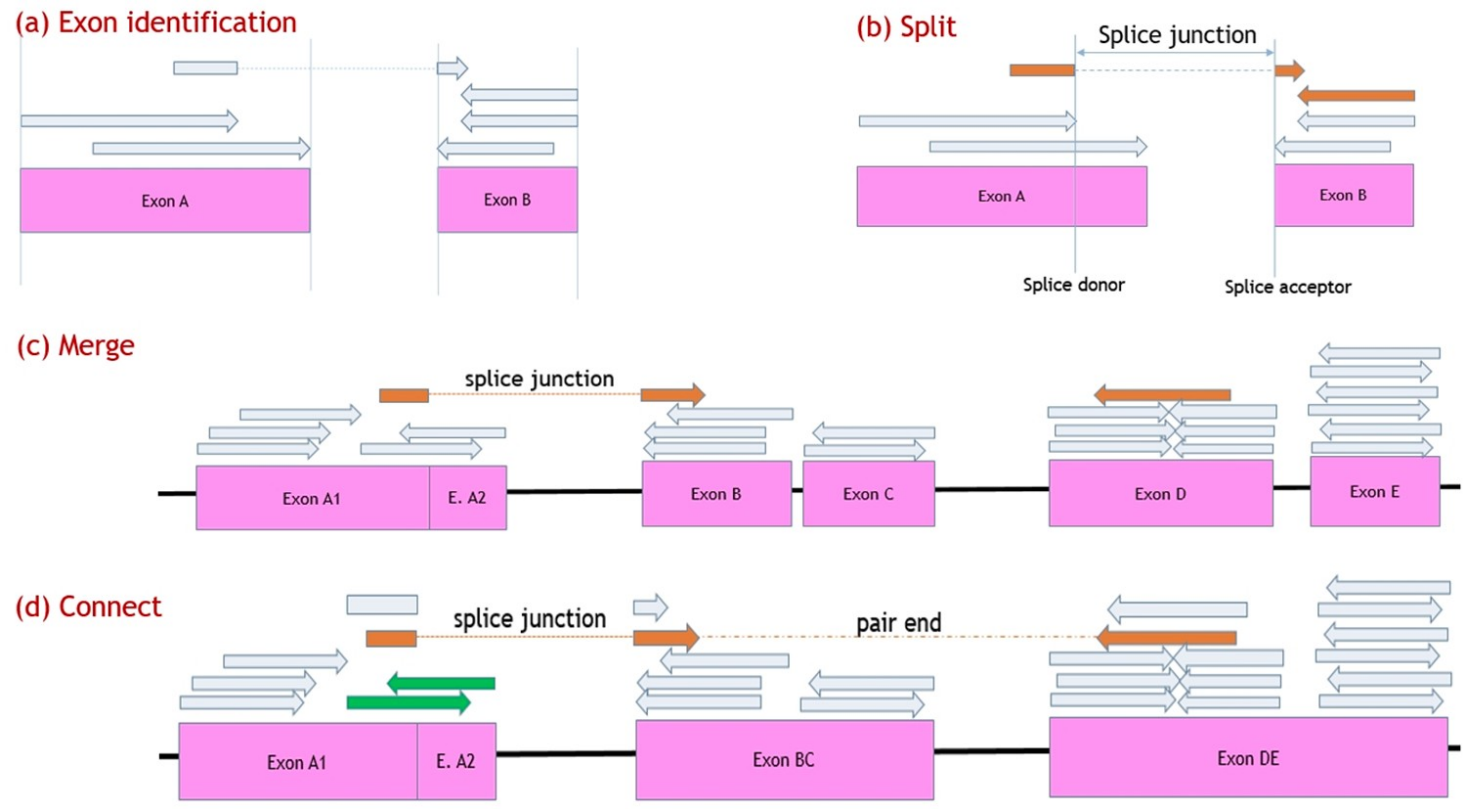
Illustrations of splice graph generation. (A) Exon regions are identified based on read alignments. (B) A splice event happens in the middle of Exon A, which implies Exon A should be further split into two sub exons at splice donor location. (C) Exon A1 and A2 should not be merged due to a splice event after A1. Exon B and C shall be merged if their gap is small. Exon D and E shall be merged if their gap is moderate but their coverage is high. (D) Exon A1 and A2 are connected because there’re read alignments (green color) crossing them. Exon A1 and BC are connected because there’re splice junctions (brown color) between them. Exon BC and DE are connected because they each contain one side of read pair alignment (brown color) and there are no other exon regions between them. A known path (A1, BC, DE) is collected because the read pair alignment (brown color) involves these three nodes.

In the split step (Fig 5B), we divide inferred exon regions (Fig 5A) into sub exon regions where splice junctions may occur. Splice junctions represent the exon or sub-exon boundaries where alternative splicing could occur. They can be determined according to the read alignments, because a read sampled across two exon parts in a transcript can be split aligned onto disconnected genome regions, with the locus where the read leaves as the splice donor and the locus where the read enters as the splice acceptor. For example, a 100-bp read could be split-aligned onto the genome (chromosome 15) at loci [78837259, 78837318] and loci [78837519, 78837558], then we consider there is a splice junction at locus 78837318 (as splice donor) and at locus 78837519 (as splice acceptor). We determine a splice junction if there’s at least one supporting alignment. This is a loose criteria and may bring false edges in the splice graph. However, this is alleviated by the next step of flow decomposition because such edges have low weights and usually will be ignored as there can be no flow decomposed to go through these edges. Once we determine a splice junction (splice donor or acceptor) in the middle of an exon, we need to divide the exon further into sub exons, because the splice junction indicates that a divided sub exon could link to another non-adjacent (sub) exon that may belong to the same RNA transcript.

In the merge step (Fig 5C), we empirically merge two exon regions based on their gap and expression levels in order to reduce the chances that the gap is actually part of exon but not covered by reads. For example, we merge two exon regions if they are very close to each other. Statistics have shown that less than 0.01% of introns are smaller than 20 bp in length [41], so a gap within 10 bp is highly unlikely intronic but should be an uncovered exonic region. We also merge two exon regions if they are moderately close to each other and have high read coverages. However, all merge procedures require two regions have no splice donors or acceptors between them, otherwise the genome gap between them should not occur together with the two regions in true transcripts.

In the connect step (Fig 5D), we establish weighted edges among nodes and collect known path information.

Two nodes are connected by a directed edge if there is a read alignment crossing one node to the other. The two nodes can be continuous, or discontinuous on the genome when there’s a splice junction between them. The related edge weight is proportional to the number of crossing read alignments. Another situation to augment edges may occur only for pair end reads. As illustrated in Fig 5D, if a read pair alignment has its first segment onto one node and the second segment onto another node, and there’re no other nodes between them, these two nodes should be adjacent in some true transcript and hereby an edge should be added to connect them, even if there’re no read alignments crossing these two nodes. A similar idea has also occurred in Scripture [15]. The weight for the augmented edge here is proportional to the number of related read pair alignments.

A known path is a sequence of nodes inferred from read alignments. Known paths are collected when a read alignment crosses more than two nodes, or a read pair alignment for an augmented edge involves more than two nodes (also illustrated in Fig 5D). We store known paths as a tuple of triple nodes. This not only provides sufficient extra information for flow decomposition later, but also helps reduce memory.

### Sparse flow decomposition

Sparse flow decomposition has been proposed in [24] for de novo assembly and here we modify it into the RefShannon framework for genome-guided assembly. Given a splice graph, it finds the minimum set of flows (e.g. paths with weights) that explains the splice graph.

Mathematically, given graph *G* = (*V*, *E*), we need to find argmin_*T*_|*T*| such that ∀*v* ∈ *V*, ∀*e* ∈ InEdges(*v*)∪OutEdges(*v*), *w*_*e*_ = ∑_*t*∈*T*,*e*∈*t*_ *f*(*t*) where *t* ∈ *T* is a path in *G* corresponds to an RNA transcript and *f*(*t*) represents the flow weight of *t*. This is a hard task, because there can be |*P*| = 2^|*V*|^ paths, and thus 2^|*P*|^ possible sets of flows.

**Fig 6:**
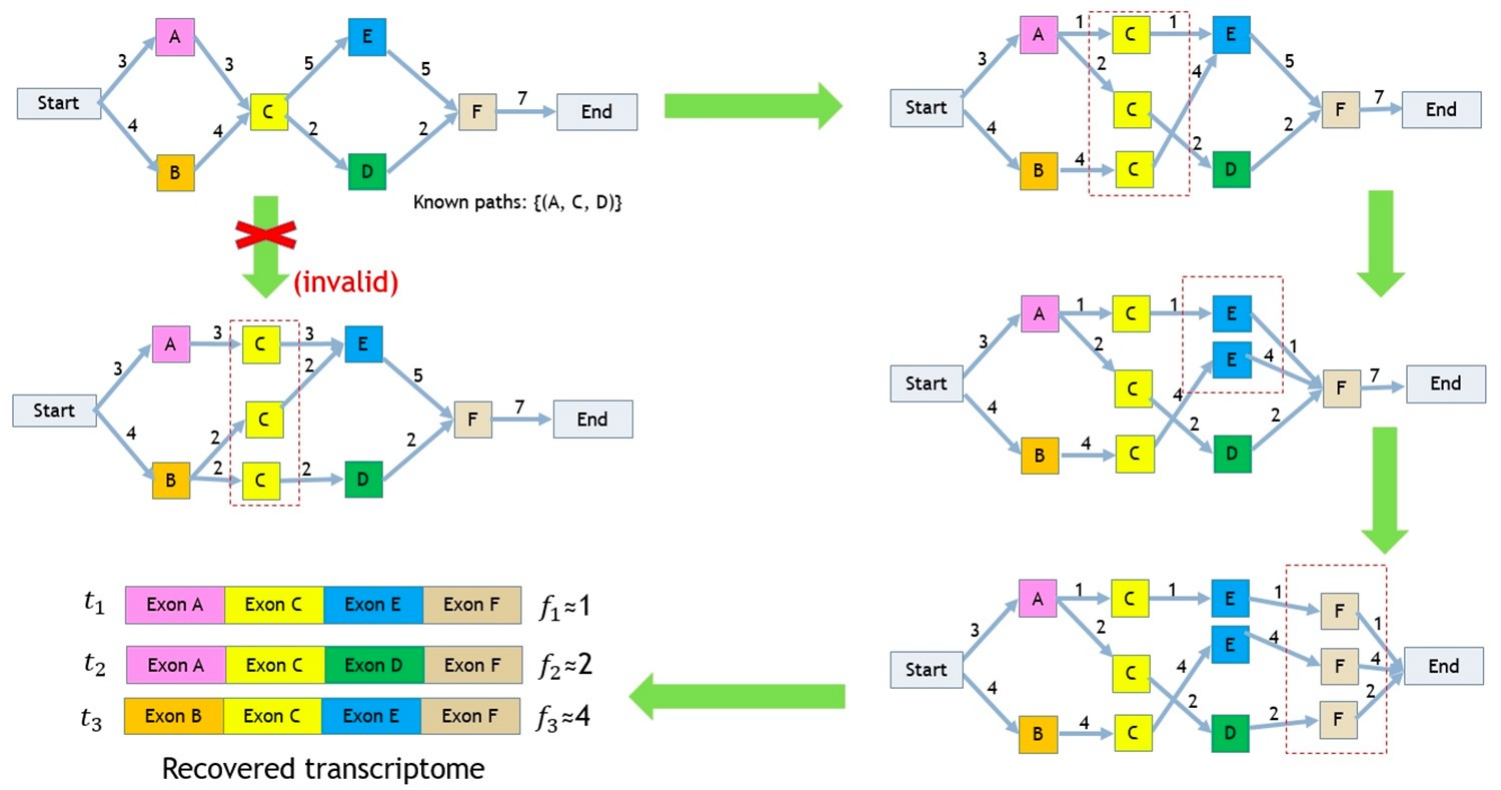
Example of sparse flow decomposition. In this figure, starting from the initial splice graph on the top left side, we try to recover transcriptome by doing local decomposition for node C,E and F iteratively.

Instead, we try to decompose a set of flows out of the splice graph node by node in topological order, as illustrated in Fig 6 (For more details, please refer to Algorithm2 and Algorithm3 in S2 File). Specifically, we first add a special source node to connect all nodes without in-edges and a special sink node to connect all nodes without out-edges. We then do local sparse flow decomposition iteratively for each node. Finally we collect flows that start from source node and stop at sink node as reconstructed RNA transcripts.

The local sparse flow decomposition at node *v* tries to find the minimum number of flows through *v* restricted by edge weight constraints. It can be formally described as: find argmin_*f*_∥*f*∥_0_ such that ∑_*i*∈InEdges(*v*)_ *f*_*i*,*j*_ = *w*_*j*_, ∀*j* ∈ OutEdges(*v*) and ∑_*j*∈OutEdges(*v*)_ *f*_*i*,*j*_ = *w*_*i*_, ∀*i* ∈ InEdges(*v*) and *f*_*i*,*j*_ ≥ 0. Here ∥*f*∥_0_ means the support set of {*f*_*i*,*j*_}, or the number of positive *f*_*i*,*j*_s.

Solving this problem may not be practically efficient. Consider for each node *v*, we have *m* × *n* possible flows (*m* = |InEdges(*v*)|, *n* = |OutEdges(*v*)|). We need to enumerate all 2^mn^ path combinations to figure out which combination offers us min ∥*f*∥_0_ and keeps the edge weight constraints.

One possible thought to ease the problem, as commonly used in signal processing, is to approximate ∥*f*∥_0_ by ∥*f*∥_1_, which unfortunately turns out to be a constant: ∥*f*∥_1_ = ∑_*i*,*j*_ *f*_*i*,*j*_ = ∑_*i*_*w*_*i*_ = ∑_*j*_*w*_*j*_. Meanwhile, we have noticed that ∥*f*∥_0_ = ∥*f* ⊙ *r*∥_0_, *r* = {*r*_*i*,*j*_|*r*_*i*,*j*_ > 0}(⊙ is element-wise product). So instead of approximating ∥*f*∥_0_ by ∥*f*∥_1_, we try to approxiate ∥*f*∥_0_ by ∥*f* ⊙ *r*∥_1_ = ∑_*i*,*j*_ *f*_*i*,*j*_ *r*_*i*,*j*_.

Therefore, we have relaxed the original problem as: find argmin_*f*_∑_*i*,*j*_ *f*_*i*,*j*_ *r*_*i*,*j*_ such that ∑_*i* ∈ InEdges(*v*)_ *f*_*i*,*j*_ = *w*_*j*_, ∀*j* ∈ OutEdges(*v*) and ∑_*j* ∈ OutEdges(*v*)_ *f*_*i*,*j*_ = *w*_*i*_, ∀*i* ∈ InEdges(*v*) and *f*_*i*,*j*_ ≥ 0, *r*_*i*,*j*_ > 0. We could use linear programming (e.g Python CVXOPT package (http://cvxopt.org/)) to solve the above problem. Since the result may contain noise, we also need to do some thresholding to get a sparse solution. To get the sparsest result, we generate *r* by a number of times and select the sparsest *f* as the final local sparse flow decomposition solution. An illustration of why the solution tends to be sparse is provided in Fig 3 in S2 File.

It is also possible that the local sparse flow decomposition may bring two results that have the same lowest sparsity and satisfy the edge constraints. This can be resolved if one of them includes a known path, as illustrated in Fig 6 as well as Fig 2C.

## Supporting information

S1 File Compare Shannon with StringTie. (PDF)

S2 File RefShannon algorithm details. (PDF)

S3 File Additional comparisons among different assemblers. (PDF)

S4 File Parameter setting for different assemblers. (PDF)

S5 File Different thresholds on sensitivity and false positive. (PDF)

S6 File Comparison of assembly performance (ROC) using different aligners. (PDF)

S7 File Compare memory and time consumption of RefShannon to other assemblers. (PDF)
