# Supplementary material for: RefShannon: A genome-guided transcriptome assembler using sparse flow decomposition
Source: PLoS One. 2020 Jun 2;15(6):e0232946. doi: 10.1371/journal.pone.0232946 (PMC7266320; doi:10.1371/journal.pone.0232946)

Supplementary File 1: Compare Shannon with StringTie

Figure 1 comes from previous experiments of (de novo) Shannon (instead of RefShannon in this paper). The dataset used is HESC (50-bp SE reads). It shows that though Shannon is a de novo transcriptome assembler, it recovers even more transcripts than state-of-art genome-guided transcriptome assembler StringTie, especially for transcripts of high read coverage. However, for transcripts of low read coverage, StringTie still reaches better sensitivity because the k-mer graph utilized by Shannon needs a higher coverage in order to stay connected.

Figure 1: **Compare Shannon with StringTie**

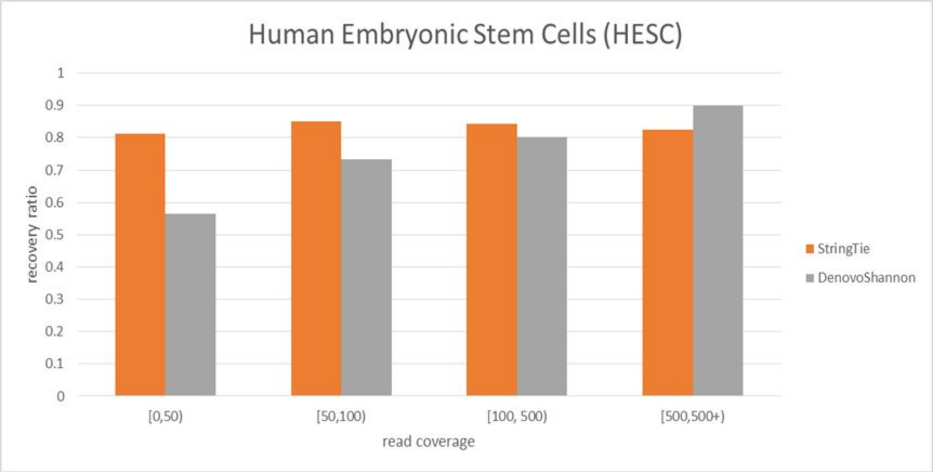

To understand this better, Figure 2 gives an example using alphabet sequences. As (a) shows, we have three reads sampled from target sequence "ABCDEF". If we build k-mer (k=3) graph from these reads, the target sequence can be sucessfully reconstructed. However, if we have insufficient reads (e.g. low read coverage) as in (b), though the target sequence is fully covered, the resulting k-mer graph is disconnected and thus we are unable to reconstruct the target sequence. On the other hand as in (c), if we utilize external knowledge of reference genome (though may not be totally same as the target one), we could align the reads onto reference genome and obtain the correct reconstructed sequence. This may explain why StringTie does better than Shannon in low read coverage region, which motivates us to start RefShannon to utilize reference genome.

Figure 2: **Example of Fragmented Kmer Graph, and Reference Genome Benefit**

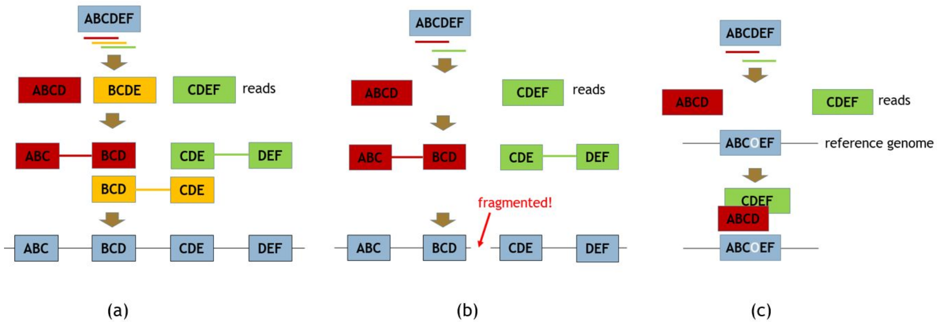

Supplement: S1 File — (PDF) [file pone.0232946.s001.pdf]
