# Supplementary material for: RefShannon: A genome-guided transcriptome assembler using sparse flow decomposition
Source: PLoS One. 2020 Jun 2;15(6):e0232946. doi: 10.1371/journal.pone.0232946 (PMC7266320; doi:10.1371/journal.pone.0232946)

Supplementary File 2: RefShannon Algorithm Details

1 Overall Flow

Figure 1 shows a more implementation-based (rather than concept-based) overall flow of RefShannon.

Figure 1: **Overall Flow of RefShannon**. Note SE means single end, PE means pair end. sam file is a file format to store read alignment. P_i is set of known paths associated with splice graph G_i, which can be further segmented into sub splice graphs G_{ij} with associated known paths P_{ij}. T_{ij} is the reconstructed transcriptome for G_{ij}. Dark blue blocks are where parallization applies.

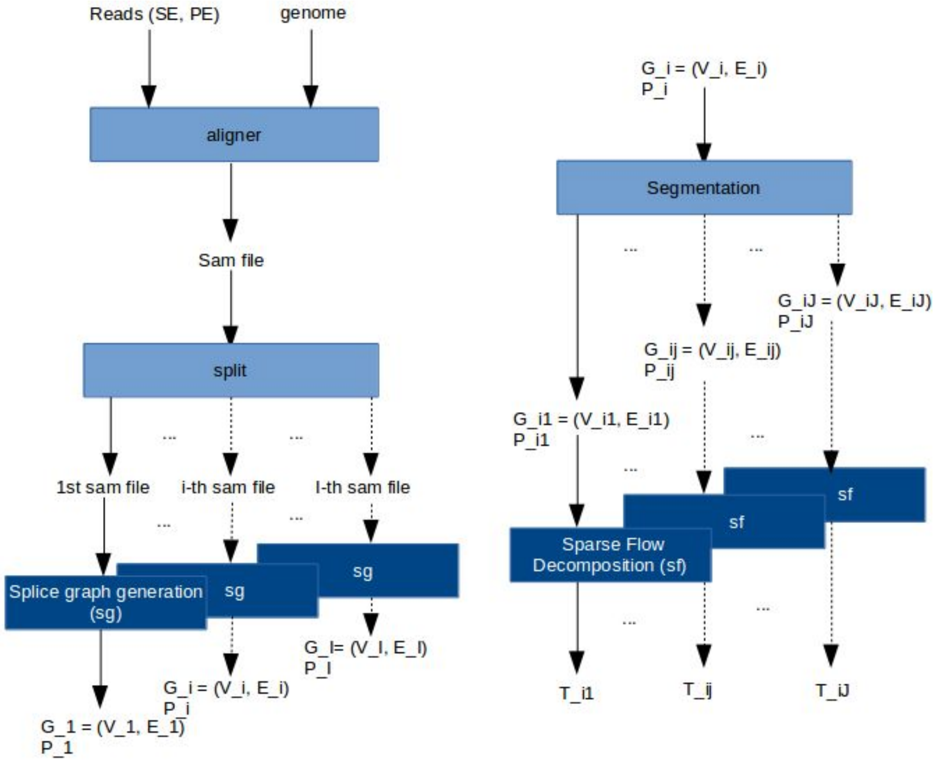

We first use alignment softwares to align reads that are either single end (SE) or pair end (PE) reads onto genome to obtain read alignment that is usually stored in sam file format (`https://samtools.github.io/hts-specs/SAMv1.pdf`). A genome can contain multiple chromosomes where DNA sequences are packed in (e.g. human genome hg19 contains chromosomes chr1, ..., chr15,...,etc).

We then split read alignment based on target chromosomes. For each smaller alignment (e.g. i-th sam file) targeted at a particular chromosome (e.g. i-th chromosome), we build a splice graph (e.g. $G_i = (V_i, E_i)$). We segment it further (as illustrated in Figure 1 and Figure 2) into (sub) splice

graphs (smaller connected components) $G_{i1} = (V_{i1}, E_{i1}), ..., G_{iJ} = (V_{iJ}, E_{iJ})$. We could consider each (sub) splice graph G_{ij} as j-th gene region on i-th chromosome, and the transcripts recovered from G_{ij} (i.e. denoted as T_{ij} in Figure 1) are j-th gene's isoforms.

We then apply a sparse flow decomposition algorithm on every (sub) splice graph G_{ij} for transcript recovery.

Parallelization (e.g. multiprocess in Python) is used in splice graph generation for different chromosomes and also used in sparse flow decomposition for different G_{ij}s within each i-th chromosome.

Figure 2: **Segmentation Example**. G is segmented into two connected components G_1 and G_2.

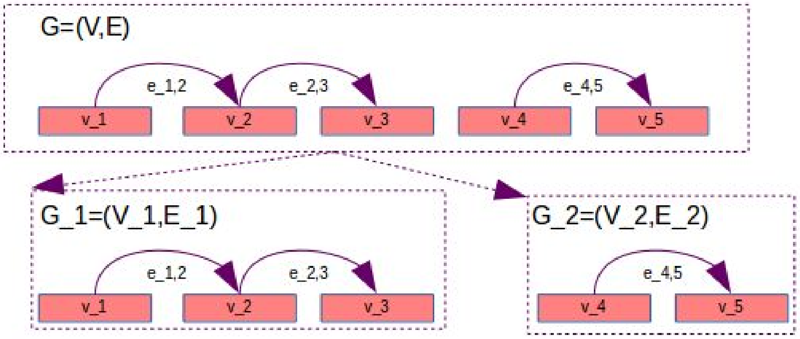

Segmentation example: G=(V,E) is segmented into G_1 and G_2

2 Splice Graph Generation

Algorithm 1 is a pseodo code of our splice graph generation method based on read alignment using pair end reads. Modification to use single end reads is straight forward.

Algorithm 1: Splice Graph Generation for Pair End Reads

Input: Set of read alignments $R^A = \{r_i^A\}$ and $R^B = \{r_i^B\}$ where (r_i^A, r_i^B) form i-th pair read alignments, as well as ROC tuning parameter $F \in [0, 1]$.

Output: Splice graph $G = (V, E)$ and known paths P_{known}. $v \in V$ refers to a genomic region (e.g. exon or a sub part of exon), $e = (v_1, v_2) \in E$ means there's flow from v_1 to v_2. Edge weight is also recorded and described by w_e. $p \in P_{known}$ is a tuple of triple nodes that is traversed by some known flow.

Infer read coverage c from read alignment $R^A \cup R^B$.

Infer splicing information s (splice junctions, splice donors and acceptors) from $R^A \cup R^B$. This could be inferred from the CIGAR pattern in sam file format of each read alignment.

Build initial set of (sub) exon regions $V^{(0)} = \{v^{(0)}\}$ from c and s.

Merge (sub) exon regions from $V^{(0)}$ to get V (i.e. node set V in splice graph $G = (V, E)$):

Init $V = \emptyset$

for $v_i^{(0)} \in V^{(0)}$ **do**

  merge $v_{i-1}^{(0)}$ and $v_i^{(0)}$ into $v_i^{(0)}$ if necessary:

  if *(1)* $Gap(v_{i-1}^{(0)}, v_i^{(0)}) \leq T_{small\_gap}$ *or (2)* $Gap(v_{i-1}^{(0)}, v_i^{(0)}) \in (T_{small\_gap}, T_{mid\_gap}]$ *and* $v_{i-1}^{(0)}$ *and* $v_i^{(0)}$ *have coverage sum* $\in [T_a, T_b(F)]$ **then**

    merge $v_{i-1}^{(0)}$ and $v_i^{(0)}$ into $v_i^{(0)}$

  otherwise, $V \leftarrow V \cup \{v_{i-1}^{(0)}\}$

$V \leftarrow V \cup \{v_{remained}^{(0)}\}$

Build edge set E and known path set P_{known} as following:

Init $E = \emptyset, P_{known} = \emptyset$

for *pair* $r_i^A \in R^A, r_i^B \in R^B$ **do**

  find r_i^A goes through regions $v_{a_1}, ..., v_{a_m}$

  find r_i^B goes through regions $v_{b_1}, ..., v_{b_n}$

  $E \leftarrow E \cup \{e_j = (v_{a_j}, v_{a_{j+1}})\}$, also update w_{e_j} for $j \in \{1, ..., m-1\}$

  $E \leftarrow E \cup \{e_k = (v_{b_k}, v_{b_{k+1}})\}$, also update w_{e_k} for $k \in \{1, ..., n-1\}$

  $P_{known} \leftarrow P_{known} \cup \{(v_{a_j}, v_{a_{j+1}}, v_{a_{j+2}})\}$ for $j \in \{1, ..., m-2\}$

  $P_{known} \leftarrow P_{known} \cup \{(v_{b_k}, v_{b_{k+1}}, v_{b_{k+2}})\}$ for $k \in \{1, ..., n-2\}$

  (assume $v_{a_1} < v_{b_1}$, or we can swap r_i^A and r_i^B)

  if *there is no* $v \in V$ *s.t.* v *is between* v_{a_m} *and* v_{b_1} **then**

    $E \leftarrow E \cup \{e = (v_{a_m}, v_{b_1})\}$, also update w_e

    $P_{known} \leftarrow P_{known} \cup \{(v_{a_{m-1}}, v_{a_m}, v_{b_1}), (v_{a_m}, v_{b_1}, v_{b_2})\}$

3 Sparse Flow Decomposition

To reconstruct transcripts from splice graph, there are both global sparse flow decomposition as well as local sparse flow decomposition. Global sparse flow decomposition iteratively does local sparse flow decomposition at each node in a topological order, while the local sparse flow decomposition tries to recover minimum number of flows under the node's inedge and outedge weight constraints. Algorithm 2 and Algorithm 3 describe the global and local sparse flow decompositions respectively.

Algorithm 2: Global Sparse Flow Decomposition

Input: A splice graph described in a direct acyclic graph $G = (V, E)$. V includes special "start" node v_s and "end" node v_e. A set of known paths P_{known}.

Output: A set T. $\forall t \in T$ is a sequence of nodes starting from v_s and ending at v_e to represent a transcript.

sort $v \in V$ in topological order
for $v \in V \setminus \{v_s, v_e\}$ *in topological order* **do**
    if v *has only 1 in-edge and 1 out-edge* **then**
        skip processing v
    else
        Prepare weights of in-edges $\{w_i, i \in InEdges(v)\}$ and weights of out-edges $\{w_j, j \in OutEdges(v)\}$ for v, and known paths for v from P_{known}
        Use them to find local sparse flow decomposition of v: $\{f_{i,j} | i \in InEdges(v), j \in OutEdges(v)\}$ (see Algorithm 3)
        for $(i, j) \in InEdges(v) \times OutEdges(v)$ **do**
            if $f_{i,j} > 0$ **then**
                create $v_{i,j}$ with same region information of v
                add $v_{i,j}$ into V
                create new edge $(InNode(i), v_{i,j})$ of weight $f_{i,j}$ into E
                create new edge $(v_{i,j}, OutNode(j))$ of weight $f_{i,j}$ into E
        remove v from V and its incident edges from E
    for $j \in OutEdges(v_s)$ **do**
        add t_j into T

Algorithm 3: Local Sparse Flow Decomposition

Input: Edge weights $\{w_i | i \in InEdges(v)\}$ and $\{w_j | j \in OutEdges(v)\}$, known paths for v: $P_{known,v} = \{p | p = (v_a, v, v_b) \in P_{known}, (v_a, v) \in InEdges(v), (v, v_b) \in OutEdges(v)\}$, as well as the ROC tuning parameter $F \in [0, 1]$

Output: A flow matrix $\{f_{i,j} | i \in InEdges(v), j \in OutEdges(v)\}$ that has the minimum number of $f_{i,j} > 0$ under constraint of $\sum_i f_{i,j} = w_j, \sum_j f_{i,j} = w_i$. $f_{i,j} > 0$ means there's a flow of weight $f_{i,j}$ going through i-th inedge of v (i.e. $InNode(i)$),v,and j-th outedge of v (i.e. $OutNode(j)$).

Init solution for output as f^{best}

for *n-th trial in N trials* **do**

- generate a coefficient matrix $r = \{r_{i,j}\}$ with $r_{i,j} = 0$ if $(InNode(i), v, OutNode(j)) \in P_{known,v}$
- get n-th solution $f^n = \{f^n_{i,j}\}$ for the Relaxed Sparse Flow Problem using linear programming:
- find $argmin_f \sum_{i,j} f_{i,j} r_{i,j}$ such that $\sum_{i \in InEdges(v)} f_{i,j} = w_j, \forall j \in OutEdges(v)$ and $\sum_{j \in OutEdges(v)} f_{i,j} = w_i, \forall i \in InEdges(v)$ and $f_{i,j} \geq 0, r_{i,j} > 0$
- trim f^n (e.g. $f_{i,j} \leftarrow 0$ if $f_{i,j} < 0.001 \times min(w_i, w_j)$)
- $f^{best} \leftarrow f^n$ if f^n is sparser than f^{best}

ROC tuning: Set $f_{ij} \in f^{best}$ to be 0 if $f_{ij}/max_{f_{ij} \in f^{best}} f_{ij} < F$

return f^{best}

3.1 Intuition of Sparse Solution

Figure 3 has given a simplified example to illustrate why the Relaxed Sparse Flow Problem (see Algorithm 3) brings sparse solution. The constraint of the example is $f_1 + f_2 = w, f_1 \geq 0, f_2 \geq 0$, which leads to the line AB in the figure. Given coefficients $r = \{r_1, r_2\}$, let the objective function be $c = r_1 f_1 + r_2 f_2$. The minimum of c is reached either at point A or point B depending on the angle of the line $r_1 f_1 + r_2 f_2$. When we try multiple coefficients r, a sparse solution thus always tends to be returned.

Figure 3: **An Example for Sparse Solution Illustration**

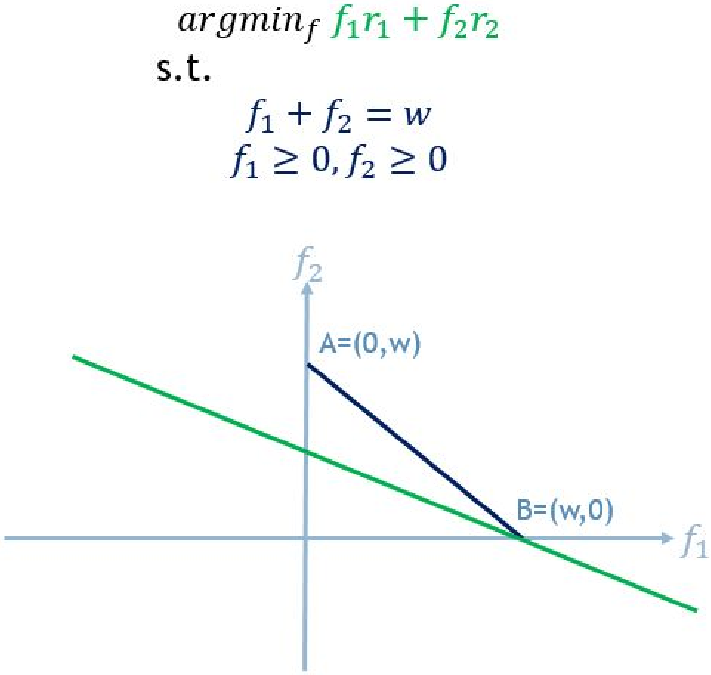

4 ROC Tuning

ROC tuning is mainly used to check the sensitivity and false positive trade off when we evaluate assemblers' performance using simulated datasets, where the ground truth reference transcripts are known.

To begin with, We notice that the merge step in splice graph generation can fill the exonic gaps but risk to increase the chances of introducing false positives. We also notice that the local sparse flow decomposition may produce noisy flows: when the sparsity of the flow set (e.g. $||f||_0$) is high, there may also be more false positive flows.

To control the sensitivity and false positive trade off, we use a threshold $F \in [0, 1]$ to adaptively adjust the merge step of splice graph generation as well as the local sparse flow decomposition output.

Specifically, when we do merge step during splice graph generation, the criteria $T_b(F)$ (below line 7 in Algorithm 1) for two regions to merge is affected by F. When F increases, $T_b(F)$ decreases to reduce region merge

operations in order to reduce false positives, accompanied by decreased sensitivity. The $T_b(F)$ is empirically determined as shown in Figure 4. When we do local sparse flow decomposition at node v and have obtained the local sparse flow solution $f = \{f_{ij} | i \in InEdges(v), j \in OutEdges(v)\}$, we set $f_{ij} \in f$ to be zero if $f_{ij}/max_{i,j} f_{ij} < F$ (see line 8 in Algorithm 3). Therefore, when F is zero, no f_{ij} is post-processed to be set as zero and max sensitivity is reached. When F is one, only f_{ij} of max flow value is returned and thus min false positive is reached. As F increases from zero to one, sensitivity decreases, resulting in reduced false positives.

The F values we use for ROC curves in main paper are selected as $0, 0.25, 0.96$.

Figure 4: $T_b(F)$ versus F

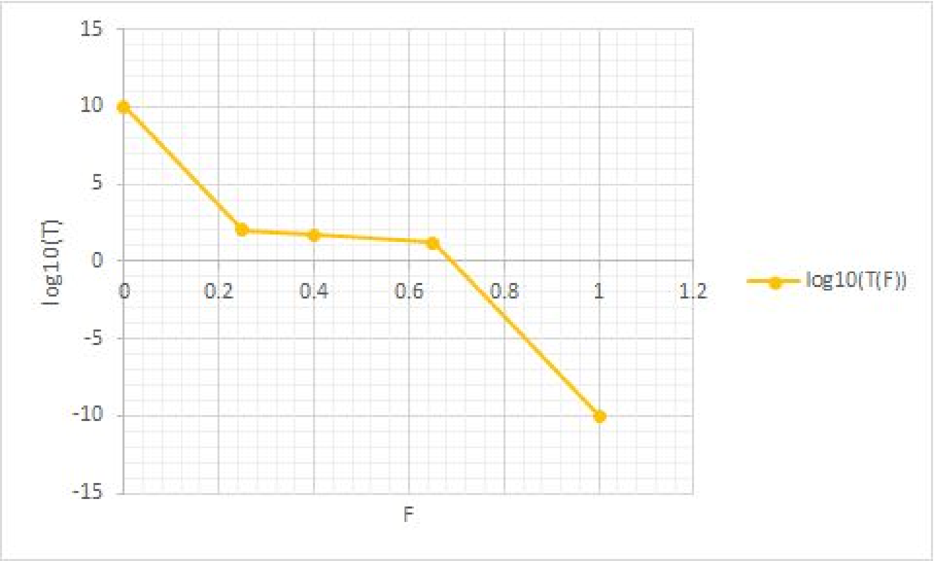

Supplement: S2 File — (PDF) [file pone.0232946.s002.pdf]
