# Supplementary material for: RefShannon: A genome-guided transcriptome assembler using sparse flow decomposition
Source: PLoS One. 2020 Jun 2;15(6):e0232946. doi: 10.1371/journal.pone.0232946 (PMC7266320; doi:10.1371/journal.pone.0232946)

Supplementary File 3: Additional Comparisons among Different Assemblers

In this supporting file, we compare RefShannon with not only popular assemblers as Stringtie (1) and Cufflinks (2), but also other genome-guided assemblers such as guided Trinity (3), Ryuto (4), Strawberry (5), TransComb (6) and CLASS2 (7). based on simulated HESC, LC and Kidney datasets aligned onto human chromosome 15 (Figure 1, Figure 2 and Figure 3). By using chromosome 15, this comparison may have smaller scale than using whole human genome (hg19) but is sufficient to show relative performances among assemblers.

Among these assemblers, RefShannon shows a better ROC operating curve with higher sensitivity at fixed false positive ratio.

In LC dataset, in addition to STAR alignments, we also use Hisat2 (Figure 2B) and Tophat2 (Figure 2C) aligners to show that the performance trend of RefShannon is not affected.

References

[1] Pertea M, Pertea GM, Antonescu CM, Chang TC, Mendell JT, Salzberg SL. StringTie enables improved reconstruction of a transcriptome from RNA-seq reads. Nat Biotech. 2015;33:290–295.

[2] Trapnell C, Williams BA, Pertea G, Mortazavi A, Kwan G, van Baren MJ, et al. Transcript assembly and quantification by RNA-Seq reveals unannotated transcripts and isoform switching during cell differentiation. Nat Biotechnol. 2010;28(5):511–515.

[3] Grabherr MG, Haas BJ, Yassour M, Levin JZ, Thompson DA, Amit I, et al. Full-length transcriptome assembly from RNA-Seq data without a reference genome. Nature Biotechnology. 2011;29(7):644–652. doi:10.1038/nbt.1883.

[4] Gatter T, Stadler PF. Ryūtō: network-flow based transcriptome reconstruction. BMC Bioinformatics. 2019;20(1). doi:10.1186/s12859-019-2786-5.

Figure 1: **Comparison of Assemblers - HESC Dataset**

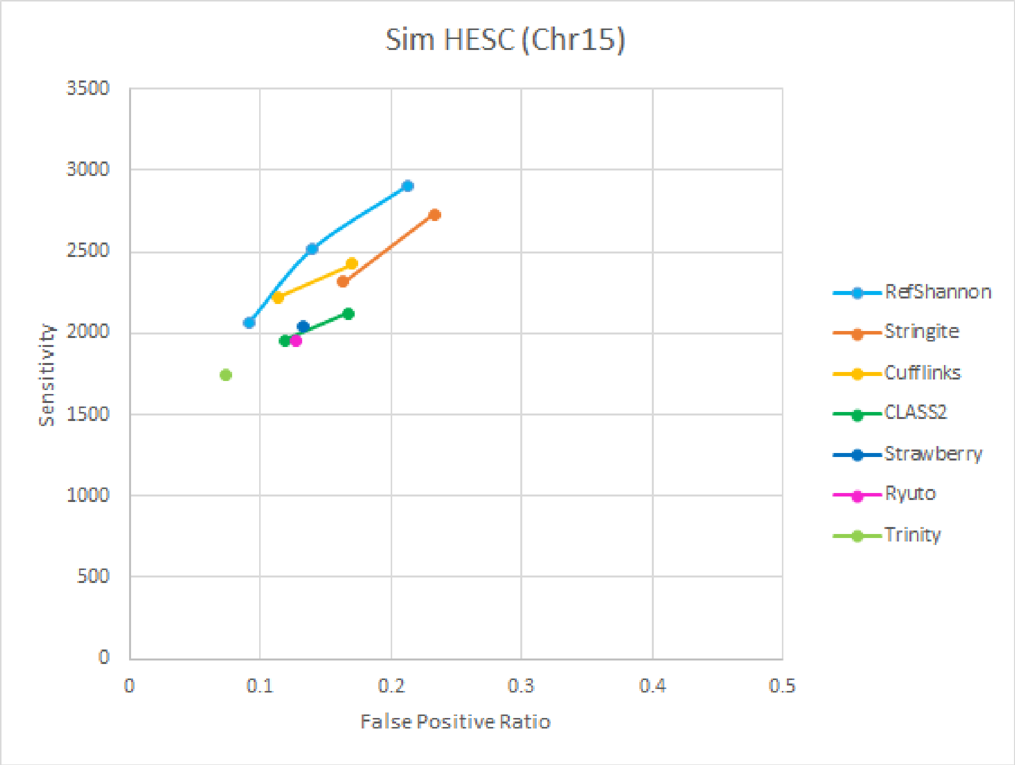

[5] Liu R, Dickerson J. Strawberry: Fast and accurate genome-guided transcript reconstruction and quantification from RNA-Seq. PLOS Computational Biology. 2017;13(11):e1005851. doi:10.1371/journal.pcbi.1005851.

[6] Liu J, Yu T, Jiang T, Li G. TransComb: genome-guided transcriptome assembly via combing junctions in splicing graphs. Genome Biology. 2016;17(1). doi:10.1186/s13059-016-1074-1.

[7] Song L, Sabunciyan S, Florea L. CLASS2: accurate and efficient splice variant annotation from RNA-seq reads. Nucleic Acids Research. 2016;44(10):e98–e98. doi:10.1093/nar/gkw158.

Figure 2: **Comparison of Assemblers - LC Dataset.** Three subplots use STAR, Hisat2 and Tophat2 respectively.

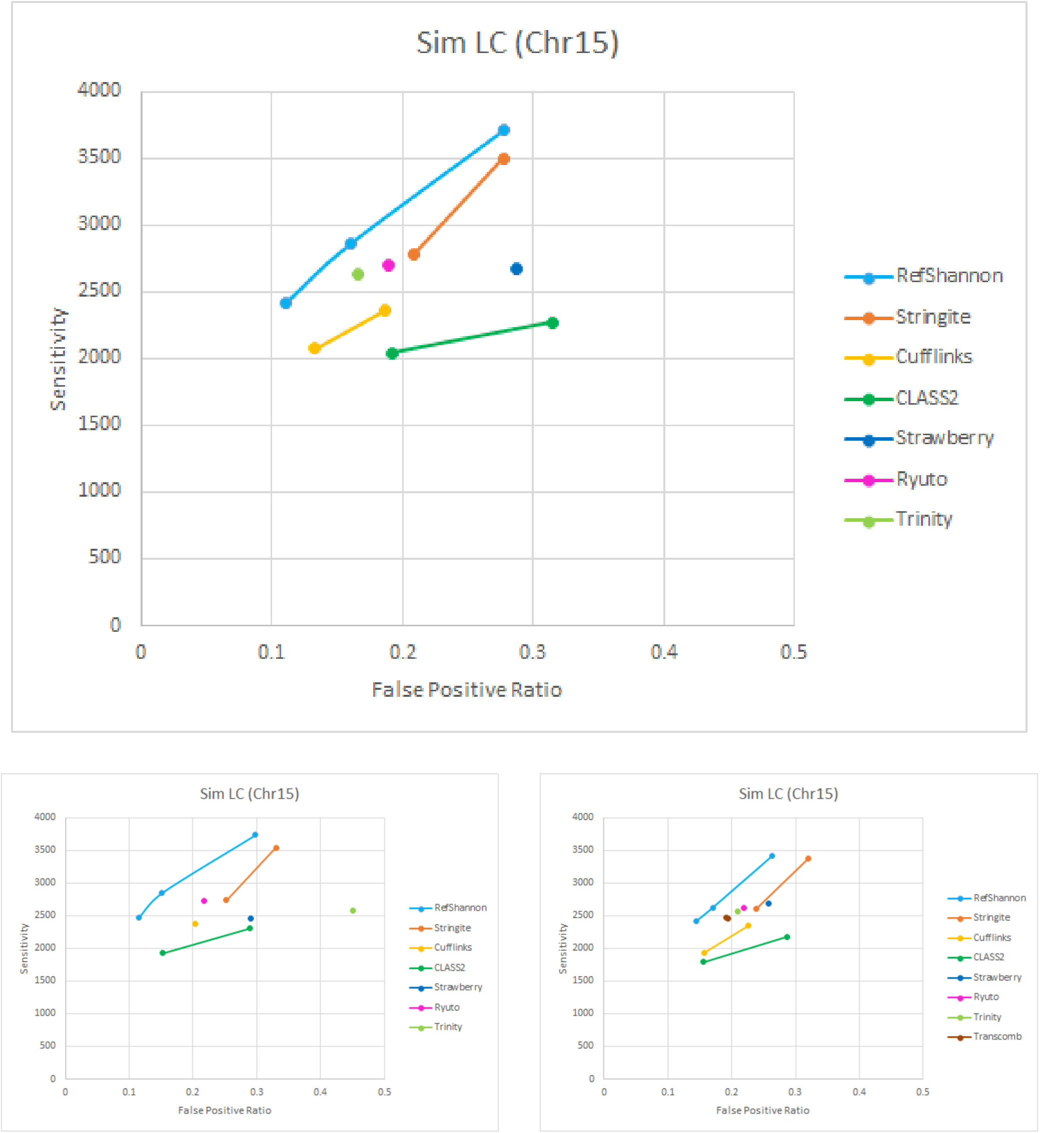

Figure 3: **Comparison of Assemblers - Kidney Dataset**

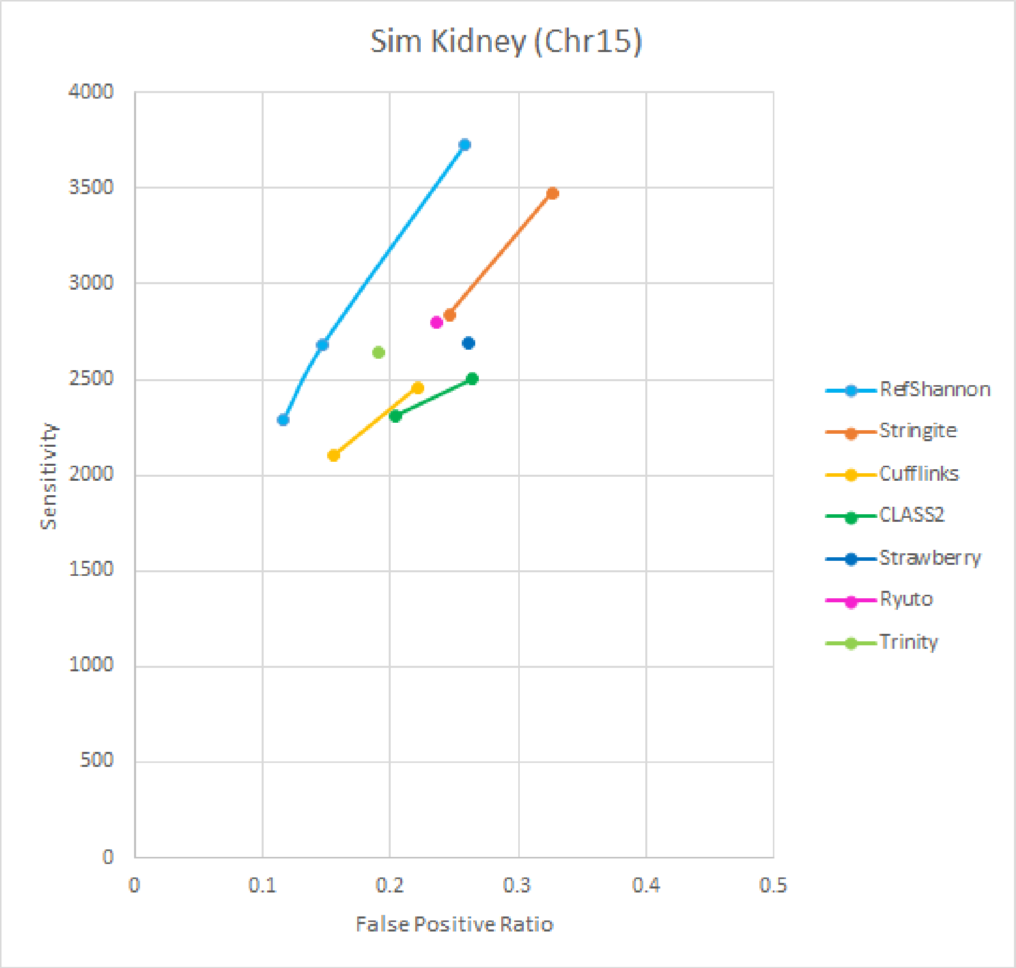

Supplement: S3 File — (PDF) [file pone.0232946.s003.pdf]
