# Supplementary material for: RefShannon: A genome-guided transcriptome assembler using sparse flow decomposition
Source: PLoS One. 2020 Jun 2;15(6):e0232946. doi: 10.1371/journal.pone.0232946 (PMC7266320; doi:10.1371/journal.pone.0232946)
Supplement: S4 File — (PDF) [file pone.0232946.s004.pdf]

Supplementary File 4: Parameter Setting for Different Assemblers

In this supporting file, we describe our setting for various assemblers including RefShannon, Stringtie (1), Cufflinks (2), guided Trinity (3), Ryuto (4), Strawberry (5), TransComb (6) and CLASS2 (7).

For RefShannon, the setting is described in Section 4 of S2 File.

For Stringtie, we use version 1.3.4d. There's default and max sensitivity mode. For max sensitivity setting of StringTie, we set "-f" as 0.0 (the minimum isoform abundance of the predicted transcripts as a fraction of the most abundant transcript assembled at a given locus, from StringTie Manual `https://ccb.jhu.edu/software/stringtie/index.shtml?t=manual`) and "-c" as 0.001 (the minimum read coverage allowed for the predicted transcripts, from StringTie Manual) in order to keep as many transcripts recovered at StringTie as possible. For StringTie, it also has another option to use super reads (e.g. longer reads) that are generated from pair end reads by external software MaSuRCA in order to improve transcript reconstruction. We have tried on simulated data but do not get performance gain for StringTie. In addition, RefShannon does not require external software to boost performance in pair end mode. Therefore, we do not include super read mode for StringTie in our performance comparison.

For Cufflinks, we use version v2.2.1. There's default and max sensitivity mode. In max sensitivity setting of Cufflinks, we set "-F" as 0.001 (the min abundance level below which transcripts are suppressed).

For guided Trinity, we use version v2.9.1 and its default mode, as several parameters such as min_kmer_cov (default 1) and genome_guided_min_coverage (default 1) seem to already have the smallest values for better sensitivity.

For Ryuto, we use version 1.3m and its default mode. We have tuned several parameters that could be related to sensitivity performance (min_filter, score_filter, no-trimming) and the sensitivity performance remains similar.

For Strawberry, we use version 1.0.2 and also its default mode because several sensitivity related parameters such as min-exon-cov (default 1) and min-depth-4-transcript (default 1) look already set for better sensitivity.

For TransComb, we use version 1.0. It only supports Tophat2 alignments. We use its default and '-f 1' settings, which are very close.

For CLASS2, we use version 2.1.7. we use its default setting, and '-F 0' setting for high sensitivity.

References

[1] Pertea M, Pertea GM, Antonescu CM, Chang TC, Mendell JT, Salzberg SL. StringTie enables improved reconstruction of a transcriptome from RNA-seq reads. Nat Biotech. 2015;33:290–295.

[2] Trapnell C, Williams BA, Pertea G, Mortazavi A, Kwan G, van Baren MJ, et al. Transcript assembly and quantification by RNA-Seq reveals unannotated transcripts and isoform switching during cell differentiation. Nat Biotechnol. 2010;28(5):511–515.

[3] Grabherr MG, Haas BJ, Yassour M, Levin JZ, Thompson DA, Amit I, et al. Full-length transcriptome assembly from RNA-Seq data without a reference genome. Nature Biotechnology. 2011;29(7):644–652. doi:10.1038/nbt.1883.

[4] Gatter T, Stadler PF. Ryūtō: network-flow based transcriptome reconstruction. BMC Bioinformatics. 2019;20(1). doi:10.1186/s12859-019-2786-5.

[5] Liu R, Dickerson J. Strawberry: Fast and accurate genome-guided transcript reconstruction and quantification from RNA-Seq. PLOS Computational Biology. 2017;13(11):e1005851. doi:10.1371/journal.pcbi.1005851.

[6] Liu J, Yu T, Jiang T, Li G. TransComb: genome-guided transcriptome assembly via combing junctions in splicing graphs. Genome Biology. 2016;17(1). doi:10.1186/s13059-016-1074-1.

[7] Song L, Sabunciyan S, Florea L. CLASS2: accurate and efficient splice variant annotation from RNA-seq reads. Nucleic Acids Research. 2016;44(10):e98–e98. doi:10.1093/nar/gkw158.
