# Supplementary material for: RefShannon: A genome-guided transcriptome assembler using sparse flow decomposition
Source: PLoS One. 2020 Jun 2;15(6):e0232946. doi: 10.1371/journal.pone.0232946 (PMC7266320; doi:10.1371/journal.pone.0232946)

Supplementary File 5: Different Thresholds on Sensitivity and False Positive

1 Thresholds to Evaluate Sensitivity Performance of Real Datasets

We first compare the sensitivity performance among different assemblers under various thresholds $T \in [0, 1]$, as illustrated in Figure 1. Note we consider each reference transcript $t \in T_{ref}$ is correctly recovered if it is over T-threshold matched with its associated assembled transcript $r \in T_{rec}$. As threshold value increases, there is a higher requirement for a reference transcript to be considered as correctly reconstructed, and thus the overall sensitivity decreases. We find throughout different thresholds and datasets, the sensitivity of RefShannon is consistently higher than StringTie and Cufflinks. Note performance gain of RefShannon over StringTie gets smaller in Kidney dataset compared to LC dataset, this can be because Kidney dataset is used in StringTie study and thus StringTie should have been optimized for this dataset. Therefore we fix $T = 0.9$ as indicated in main paper.

Figure 1: Sensitivity Performance versus Varying Thresholds. x-axis is threshold T and y-axis is sensitivity - the number of reference transcripts that are considered to be correctly reconstructed..

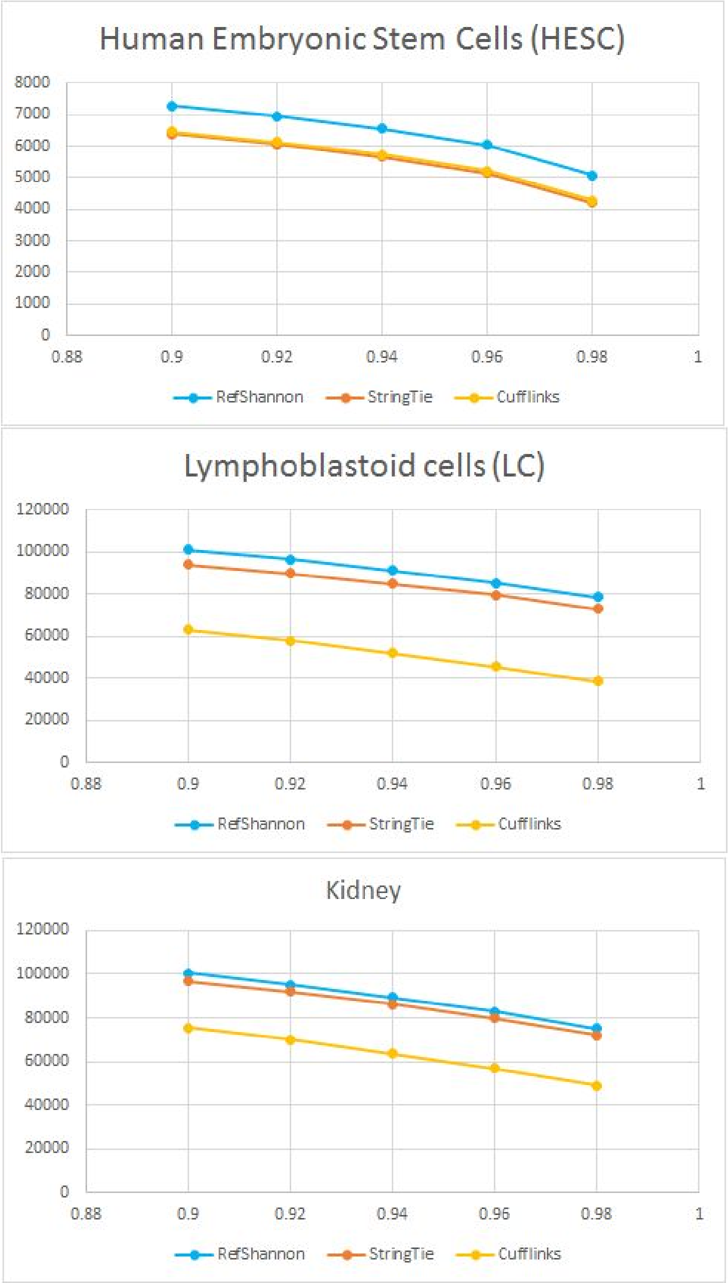

2 Thresholds to Evaluate ROC Performance of Simulated Datasets

Figure 2 shows how sensitivity changes for simulated datasets, as we vary thresholds T. Similar as in Figure 1, all assemblers are in max sensitivity setting and RefShannon still shows consistently higher sensitivity than StringTie and Cufflinks. For HESC dataset, Cufflinks does not show a similarly close performance with StringTie as it does in Figure 1, this can be because the reference transcripts used here are based on LC dataset and have more complex splice patterns. Still, Cufflinks drops more in LC and Kidney datasets (e.g. PE reads) compared to HESC dataset (e.g. SE reads), implying it may throw away pair end read alignments of uncertain compatibility.

Figure 2: Sensitivity Performance versus Varying Thresholds. x-axis is threshold T and y-axis is sensitivity..

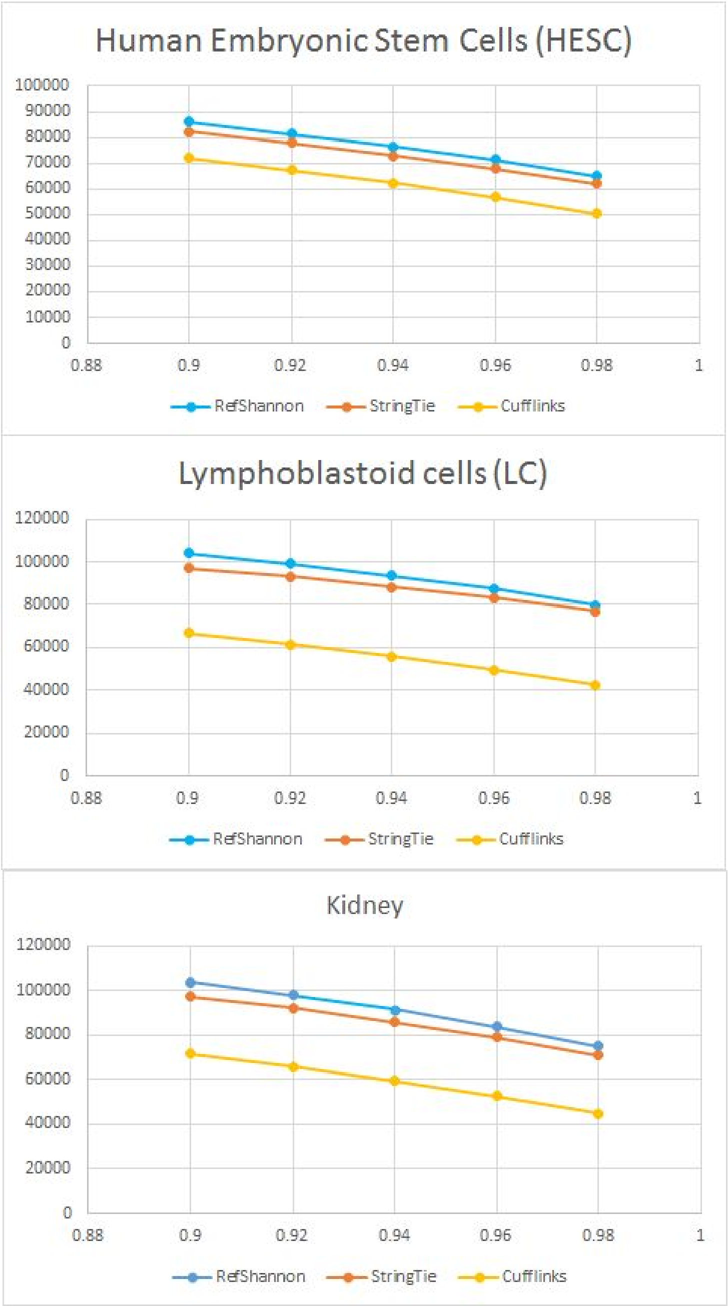

Figure 3 shows how false positive changes for simulated datasets, as we vary threshold T. Recall we consider a reconstructed transcript $r \in T_{rec}$ to be a false positive if it is below T-threshold (e.g. 90%) matched with any reference transcript $t \in T_{ref}$. As false positive threshold increases, a reconstructed transcript needs to be matched by a higher threshold with associated reference transcript to be considered as non false positive. Therefore, the overall false positive increases. Note here RefShannon is in its min false positive setting while StringTie and Cufflinks are in their default settings. It's possible that StringTie and Cufflinks may have lower false positive settings but with a decreased sensitivity trade off. Here we mainly want to show our choice of threshold T as 0.9 does not affect the relative performance among assemblers.

Figure 3: False Positive Performance versus Varying Thresholds. x-axis is false positive threshold T, and y-axis is false positive ratio.

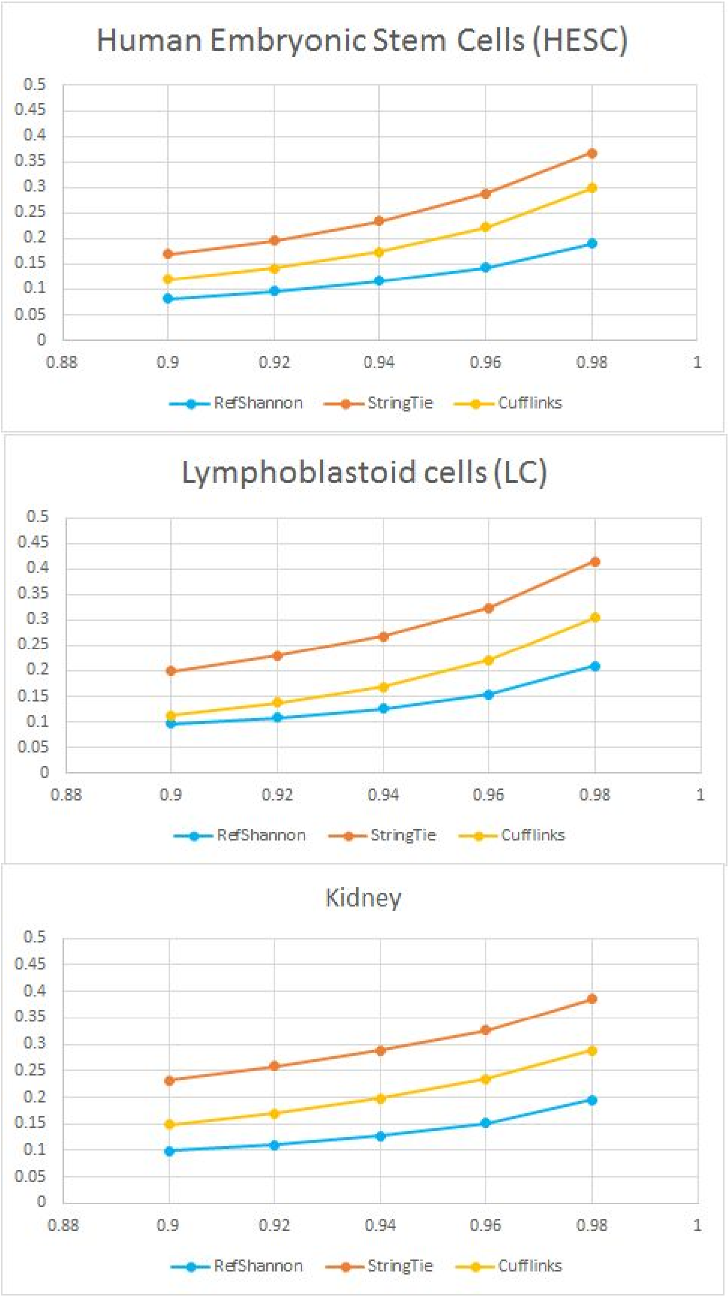

Supplement: S5 File — (PDF) [file pone.0232946.s005.pdf]
