# Supplementary material for: RefShannon: A genome-guided transcriptome assembler using sparse flow decomposition
Source: PLoS One. 2020 Jun 2;15(6):e0232946. doi: 10.1371/journal.pone.0232946 (PMC7266320; doi:10.1371/journal.pone.0232946)

Supplementary File 6: Comparison of assembly performance (ROC) using different aligners

We have compared the ROC performance of each assembler using different aligners (STAR v2.5.2a, Tophat2 v2.1.1, Hisat2 v2.1.0; both Tophat2 and Hisat2 are most updated versions), based on the simulated LC dataset (chromosome 15).

The assemblers of Stringtie, Cufflinks and CLASS2 have both default and maximum sensitivity performances. The assemblers of Ryuto, Trinity and Strawberry have their default performances, as their default parameters tend to favor better sensitivity. TransComb is not included as it only works for Tophat2.

Using the alignment results of STAR has achieved best ROC performance for most assemblers (e.g. RefShannon, Stringtie, Cufflinks, Ryuto, Trinity). Hisat2 performs better than Tophat2 for more assemblers (e.g. RefShannon, Cufflinks, Ryuto, and CLASS2). As a result, we proceed with our evaluation using the STAR aligner.

We checked the alignment patterns (i.e. FLAGS) in especially in Tophat2. Tophat2 contains more noisy flags (for instance, the flag of 'each segment properly aligned according to the aligner' is not set), and this might cause performance degradation for assemblers.

Figure 1: **Comparison of Aligners**

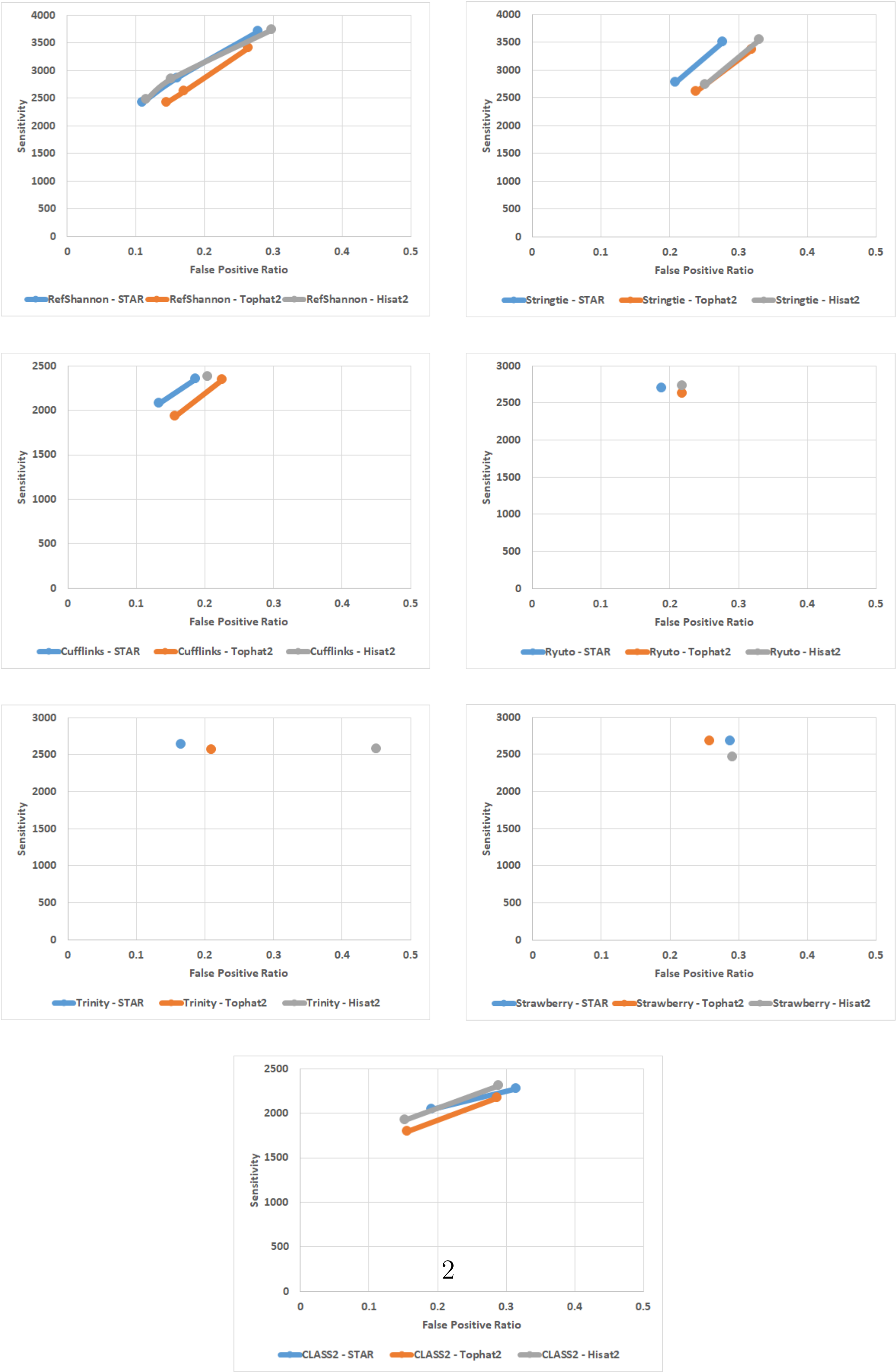

Supplement: S6 File — (PDF) [file pone.0232946.s006.pdf]
