# Supplementary material for: RefShannon: A genome-guided transcriptome assembler using sparse flow decomposition
Source: PLoS One. 2020 Jun 2;15(6):e0232946. doi: 10.1371/journal.pone.0232946 (PMC7266320; doi:10.1371/journal.pone.0232946)

Supplementary 7: Compare memory and time consumption of RefShannon to other assemblers

We have run RefShannon, StringTie, Cufflinks, Ryuto and Trinity by their default mode on the three real datasets and benchmarked their memory consumption and time consumption using cgmemtime (`https://github.com/gsauthof/cgmemtime`), which measures the high-water RSS plus CACHE memory usage of involved threads[1], as well as the total wall clock time.

To see an overall picture on the memory and time consumption by different assemblers on various datasets, we first conduct experiments using 20 CPUs (AMD Opteron 6380, 1400MHz) of a lab server with 515G total memory (Table 1 and Table 2). In terms of speed, StringTie is the fastest whereas RefShannon is overall faster than Cufflinks, guided Trinity and Ryuto. In terms of memory consumption, Stringtie and Cufflinks work better especially in the largest Kidney dataset, whereas RefShannon's memory consumption is high. Guided Trinity takes more time and memory, which is reasonable as it is essentially doing de novo assembly but in smaller regions.

Table 1: Memory Comsumption (GB)

Datasets	RefShannon	StringTie	Cufflinks	Ryuto	Trinity
HESC (132M SE reads)	59.69	0.98	7	**0.65**	100.26
LC (115M PE reads)	106.2	15	13.18	**3.56**	187.43
Kidney (183M PE reads)	166	**27.36**	30.99	70.50	281.48

Table 2: Time Comsumption

Datasets	RefShannon	StringTie	Cufflinks	Ryuto	Trinity
HESC (132M SE reads)	34.5 min	**4.3 min**	58.6 min	10.32 min	12.1 hrs
LC (115M PE reads)	5.19 hrs	**21.7 min**	8.27 hrs	5.26 hrs	30.8 hrs
Kidney (183M PE reads)	9.23 hrs	**38.37 min**	43.77 hrs	16.69 hrs	41.7 hrs

To understand in further details, we check how memory and time consumption change as the number of threads (or processes) changes. The experiments are conducted on all of the three real datasets (Fig 1 to 6). Note

[1]For RefShannon, we apply multiprocess instead of multithreads.

we only plot memory/time consumption of Trinity using 20 threads as it takes much more time to complete one assembly task.

In terms of memory, the amount RefShannon consumes is almost proportional to the number of processes, while Stringtie, Cufflinks and Ryuto consume much less. The high memory consumption of RefShannon can be because of the usage of Python and multiprocessing.

In terms of time, RefShannon is overall faster than Cufflinks and Trinity, tend to be faster than Ryuto in large dataset using more processes, but slower than Stringtie. It is somehow expected that RefShannon takes more time as it conducts more examinations on the pair end reads information especially during splice graph generation.

Currently, a typical lab server with at least 20 CPU cores and over 200GB memory would be sufficient to run RefShannon on large real datasets. We leave the time/memory optimization as our future work.

Figure 1: HESC - Memory

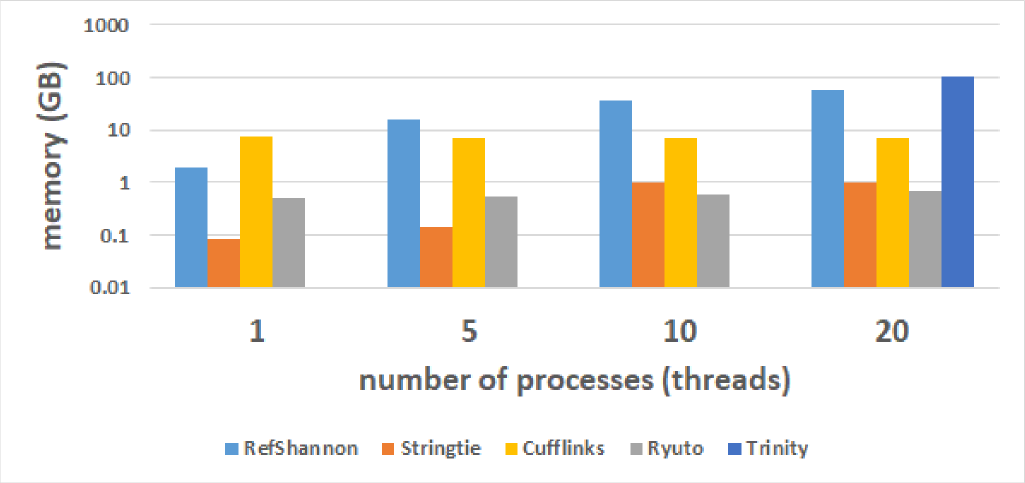

Figure 2: HESC - Time

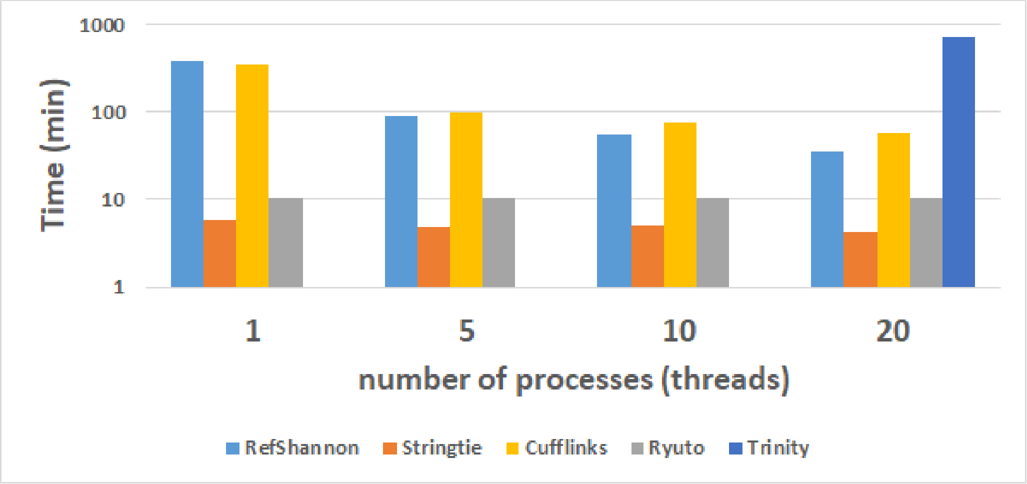

Figure 3: LC - Memory

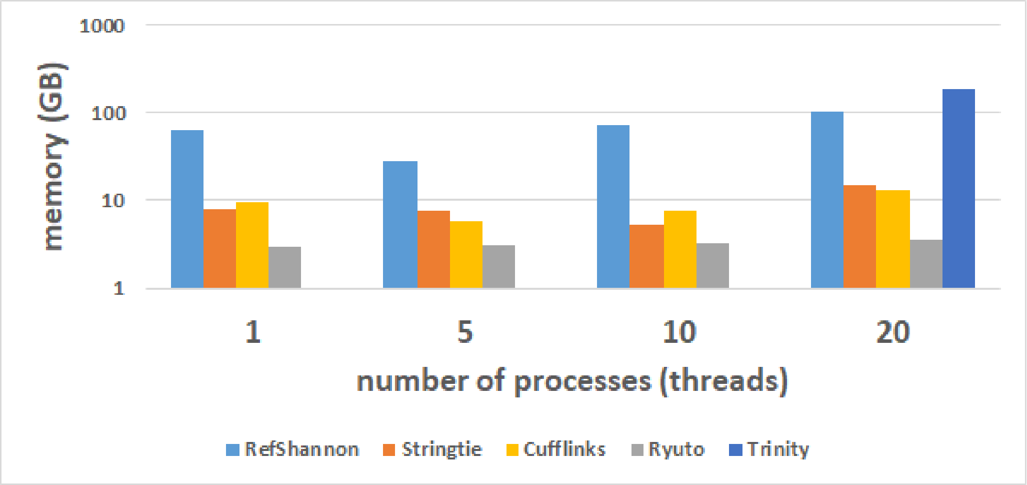

Figure 4: LC - Time

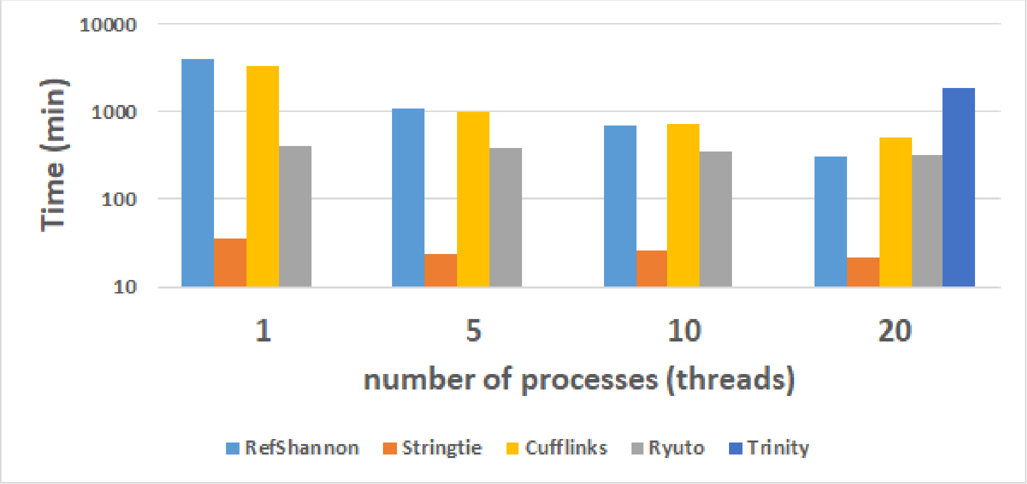

Figure 5: Kidney - Memory

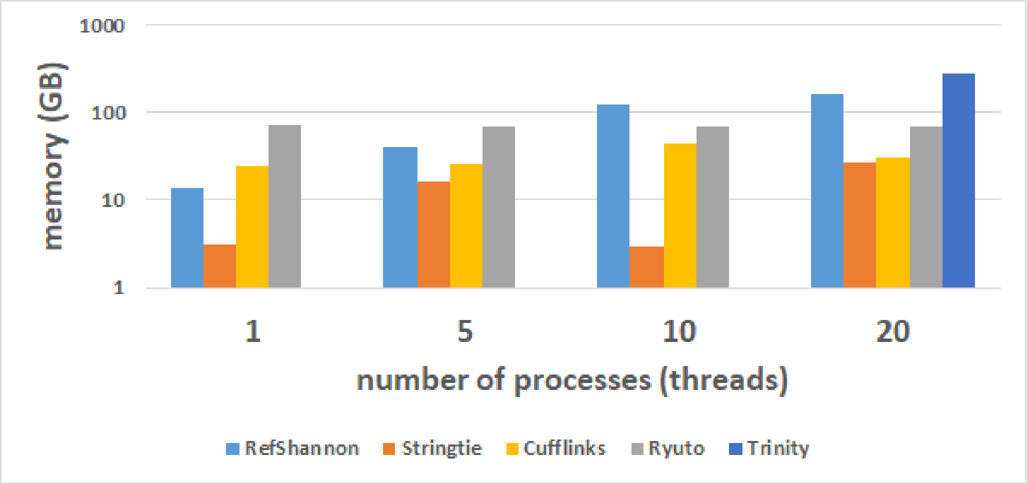

Figure 6: Kidney - Time

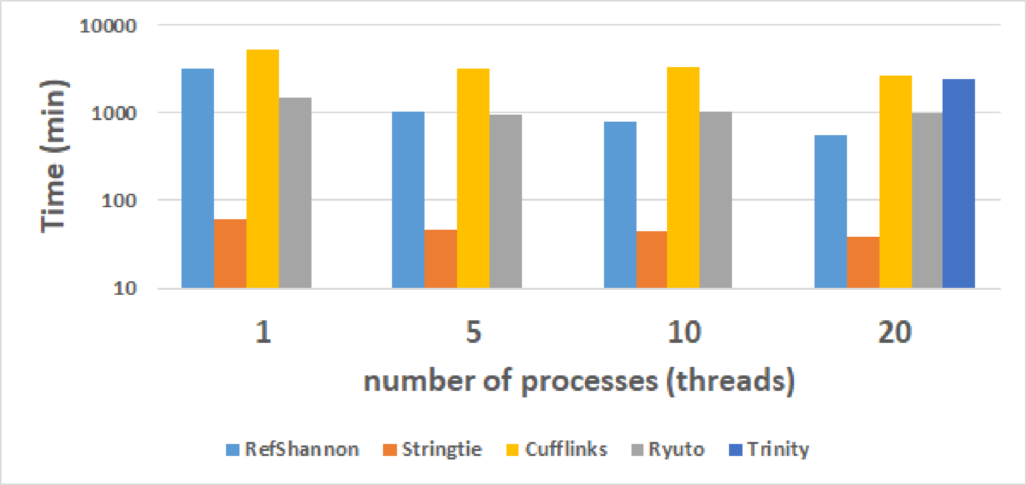

Supplement: S7 File — (PDF) [file pone.0232946.s007.pdf]
